# Enhanced carbon dioxide drainage observed in digital rock under intermediate wetting conditions

**DOI:** 10.1038/s41598-024-65920-6

**Published:** 2024-07-09

**Authors:** Jaione Tirapu Azpiroz, Ronaldo Giro, Rodrigo Neumann Barros Ferreira, Marcio Nogueira Pereira da Silva, Manuela Fernandes Blanco Rodriguez, Adolfo E. Correa Lopez, David A. Lazo Vasquez, Matheus Esteves Ferreira, Mariana Del Grande, Ademir Ferreira Da Silva, Mathias B. Steiner

**Affiliations:** 1 IBM Research, Av. República do Chile, 330, Rio de Janeiro, RJ CEP 20031-170 Brazil; 2 IBM Research, Rd J Fco Aguirre Proenca Km 9 Sp101, Hortolandia, Sao Paulo, SP 13186-900 Brazil; 3 IBM Research, Rua Tutóia, 1157, São Paulo, Sao Paulo, SP 04007-005 Brazil

**Keywords:** Fluid dynamics, Geophysics, Climate-change mitigation

## Abstract

Carbon dioxide (CO$_2$) trapping in capillary networks of reservoir rocks is a pathway to long-term geological storage. At pore scale, CO$_2$ drainage displacement depends on injection pressure, temperature, and the rock’s interaction with the surrounding fluids. Modeling this interaction requires adequate representations of both capillary volume and surface. For the lack of scalable representations, however, the prediction of a rock’s CO$_2$ storage potential has been challenging. Here, we report how to represent a rock’s pore space by statistically sampled capillary networks (ssCN) that preserve morphological rock characteristics. We have used the ssCN method to simulate CO$_2$ drainage within a representative sandstone sample at reservoir pressures and temperatures, exploring intermediate- and CO$_2$-wet conditions. This wetting regime is often neglected, despite evidence of plausibility. By raising pressure and temperature we observe increasing CO$_2$ penetration within the capillary network. For contact angles approaching 90$^\circ$, the CO$_2$ saturation exhibits a pronounced maximum reaching 80$\%$ of the accessible pore volume. This is about twice as high as the saturation values reported previously. For enabling validation of our results and a broader application of our methodology, we have made available the rock tomography data, the digital rock computational workflows, and the ssCN models used in this study.

## Introduction

To achieve global targets for mitigating greenhouse gas emission, efficient carbon capture and storage technologies are needed^1^. Captured and purified CO$_2$ can be injected into subsurface rock formations, providing a pathway to long-term geological storage at scale. Encouragingly, estimates of the connected void space in reservoir rock of suitable geological formations exceed the volume required for satisfying the global CO$_2$ storage needs^2^. The physical and chemical processes underlying CO$_2$ geological storage are: (i) structural storage under impermeable cap rocks; (ii) residual or capillary trapping; (iii) dissolution in water/brine and (iv) mineralization. Processes (i) and (ii) occur at shorter time scales—typically within a few years–but carry an elevated risk of CO$_2$ leakage^3^. Water-wet reservoir conditions lower the risk of CO$_2$ leakage in both structural and residual trapping, either by lowering the CO$_2$ permeability of the cap rock, or by raising the capillary pressures for increased CO$_2$ trapping efficiency at pore scale^4–6^. The processes (iii) and (iv) carry a lower risk of CO$_2$ leakage, however, they occur at longer time scales, ranging from decades to centuries^3^. Capillary trapping of CO$_2$ has been reported to achieve saturation levels of 10–30$\%$ of the rock’s pore volume^3^ and is a promising candidate process for low-risk CO$_2$ storage at scale.

The efficiency of trapping CO$_2$ in deep geological formations is determined by the microscopic properties of the rock’s connected pore space and depends on its interaction with supercritical CO$_2$ (scCO$_2$) and brine^3,7–16^. Extensive research has been carried out for investigating the role of surface wettability on fluid displacement^16^; the relative permeability and trapping of CO$_2$ in sandstone^8,9,11^, as well as in carbonate^10,11^, in shale and in anhydrite rocks^11^. In the case of sandstones, which are promising candidates for CO$_2$ storage at field scale^3^, experimental studies of CO$_2$ capillary trapping at reservoir conditions yielded maximum CO$_2$ saturation values of 46–59% of rock pore volume^8^. CO$_2$ is injected into subsurface porous formations during drainage, displacing the resident fluid as it migrates within the pore space of the reservoir as the non-wetting fluid in response to the pressure gradients^3,17^. While strongly water-wet reservoirs are characteristic of residual CO$_2$ trapping, the maximum saturation achievable is limited^8^. Recent studies have, therefore, investigated the potential of intermediate- and CO$_2$-wet reservoir conditions^4,5,18^, including a wide range of wettability values from strongly water-wet to CO$_2$-wet^19^. However, for determining optimum CO$_2$ storage potential conditions, the evaluation would benefit from controlled interrogation of the parameter space.

Suitable numerical modeling approaches for simulating flow in reservoir rock at pore scale include mesh- or lattice-based direct simulation methods^12,20,21^ for achieving high fidelity, and network based methods^22–24^ with improved computational efficiency. For optimizing the CO$_2$ capillary trapping in a given rock, it is necessary to screen for pressure and temperature conditions as a function of wettability, i.e., the contact angle at the interface between the rock, scCO$_2$ and brine. While prior studies have demonstrated the dependence of a rock’s CO$_2$ storage potential on both the capillary network and its surface wetting properties^16^, mapping the relevant parameter space with lab experiments in a controlled manner is impractical.

In the following, we report a new methodology to simulate CO$_2$ injection into the pore space of rock samples, based on the capillary network representation of reservoir rock, that circumvents the computational cost of computing on billions of capillaries by simulating on morphologically equivalent but more computationally efficient networks. We apply the new method to simulate CO$_2$ drainage displacement in sandstone under realistic reservoir conditions, tightly mapping the relevant parameter space for identifying conditions with optimum storage potential. The CO$_2$ retained after drainage represents the maximum amount that can remain in the pore space after imbibition, and therefore can be considered as a measure of the “storage potential”. The computational efficiency of our method to simulate two-phase flow in statistically meaningful rock sample sizes allowed the study over a large range of injection conditions, and extracting insight applicable to the reservoir scale^24^. We have unveiled a potentially more optimum regime for CO$_2$ drainage displacement under intermediate-wet conditions than previously considered. Our study also sheds light onto an emergent phenomenon resulting from the collective behavior of the capillary network leading to counterintuitive low saturation results under CO$_2$-wet conditions.

## Results and discussion

### Simplified network methodology

We have developed a pore-scale flow simulator for studying injection and saturation of porous rock samples modeled as a network of capillaries^24,25^. Specifically, we have simulated the injection of supercritical CO$_2$ into the capillary network model of a Berea Sister Gray sandstone sample filled with water as the resident fluid. Computationally, we track the displacement in time of the fluid interface between scCO$_2$ and water within each capillary of the connected pore space. To overcome the computational limitations, we have developed a sampling technique in which the assessment is performed with the aggregate result of multiple flow simulations performed with sets of much smaller but statistically equivalent capillary network models taken from the same digital rock sample. Importantly, each sub-sample matches statistically the morphlogical and geometrical properties of the original capillary network.

**Figure 1:**
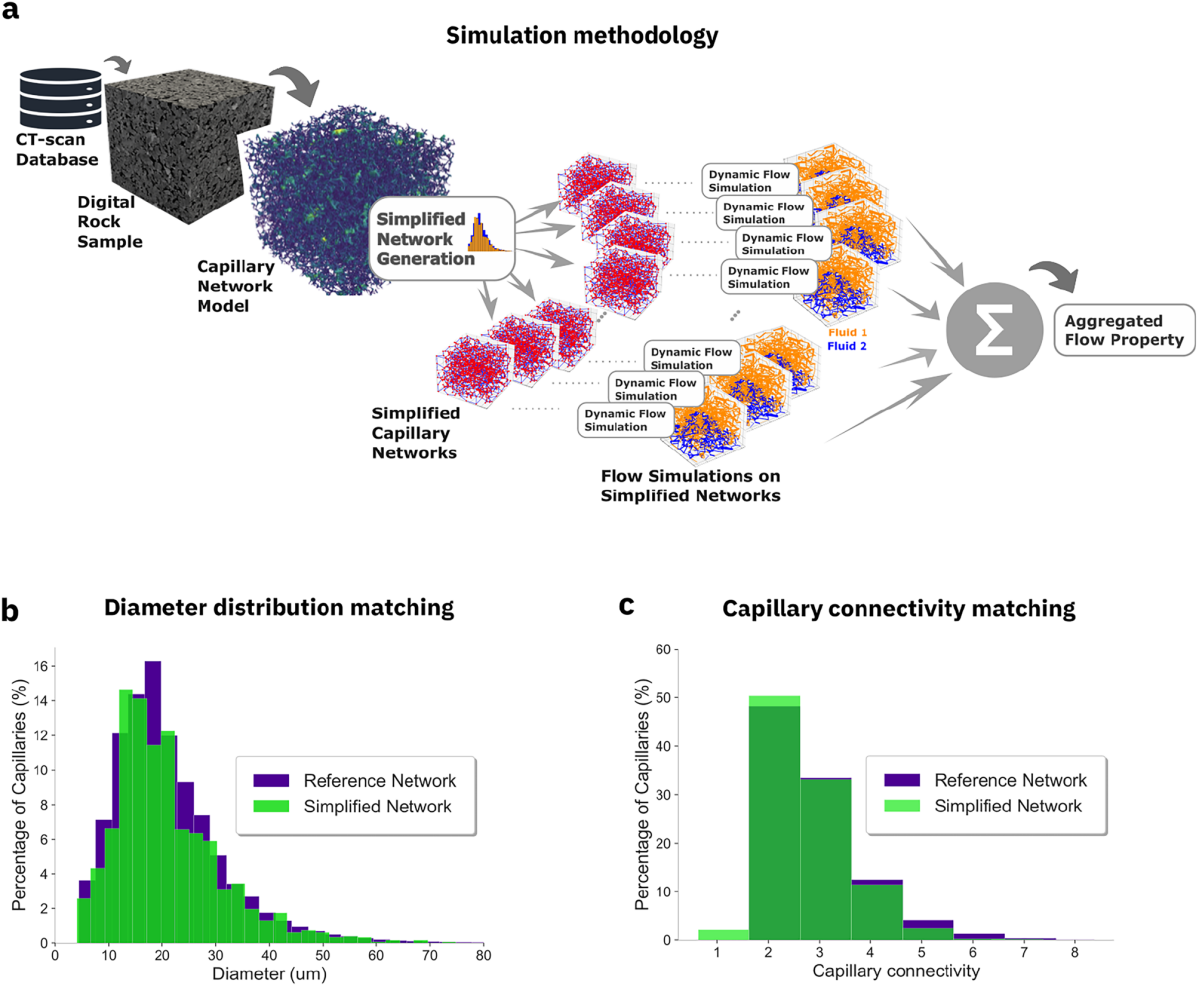
Simplified network methodology. (**a**) Schematic workflow from rock tomography to flow properties. Distribution of (**b**) capillary diameter and (**c**) capillary connectivity for the capillary network of the original Berea sandstone rock sample as a reference and a statistically simplified one for comparison.

In Fig. 1 we display the methodological workflow. As input, we have used grayscale rock images acquired with X-ray computed micro-tomography ($\mu$CT) scans^26,27^, and that we made publicly available as a digital rock dataset^28,29^. A sequence of image processing steps are applied to the image for reducing noise, increasing contrast and for separating solid and void spaces (see *Supplementary Information* Section S1and Supplementary Fig. S1). As a representative rock sample, we have chosen a sandstone referred to as “Berea Sister Gray”. We have used the Centerline algorithm to create a CNM of the binarized digital rock image^24^. The CNM extracts from the pore space a voxel-wide line at the center of the pore channels, annotated with the pore radii at each point in space. The pore space is represented as a sequence of short cylinders with gradually changing radii. Each cylinder radius is defined as the radius of inscribed circle into the pore space centered at the point belonging to the line at the center of the pore channel (more details can be found in Section *Methods* and in Section S2 of the *Supplementary Information*). An example of the resulting CNM for a REV-sized sandstone sample is shown in Fig. 1. Supplementary Fig. S3 displays the induced pressure field inside the network when an external 10 kPa/m pressure gradient is imposed along each axis.

We have built sets of statistically sampled capillary network (ssCN) representations preserving the following properties: (i) porosity; (ii) capillary diameter distribution; and (iii) node coordination number distribution. As a result, we obtain morphologically equivalent networks containing only a small fraction of the capillaries, i.e., $2.7 \times 10^6$ capillaries are reduced to $1.8 \times 10^3$ capillaries. Each simplified network in the set begins as a random distribution of nodes and capillaries. Following an optimization routine that aims to match the properties of capillary connectivity, length, diameter, and general porosity of the original rock sample, it is then transformed into a network of capillaries with equivalent morphological properties as the original one. The result is an ensemble of simplified network models with significantly reduced number of capillaries that maintains the original CNM properties.

This equivalence is seen by comparing the distributions of capillary diameters and capillary connectivities displayed in Fig. 1b,c, respectively, as percentage of the total number of capillaries between the original rock sample and the algorithmically generated one. The final step in our workflow consists of collecting the flow properties of the original rock sample from the average of the results taken from the set of *k*-ssCN simplified equivalent networks. We find that 1500 capillaries per ssCN, about $0.5\%$ of the original number, provides a good balance between computational accuracy and cost. As a result, the computed permeability averaged over an ensemble of 50 ssCN instances falls within $\pm 3 \sigma$ of the original value. Increasing the number of capillaries in the simplified representation reduces the variability of the results, however, it does not significantly improve the accuracy of the average estimate when compared to the original. More details can be found in Section S5 of the *Supplementary Information*.

### Simulation of CO$_2$ drainage displacement

Following the method outlined above, we have performed a sensitivity analysis with respect to multiple fluid parameters, such as temperature, contact angle, pressure gradient, and quantified their influence on the infiltration and retention of CO$_2$ inside an ensemble of simplified capillary networks that is representative of the target Berea Sister Gray sandstone sample. To determine under which conditions CO$_2$ drainage displacement through capillary forces is maximized, we have considered temperature scenarios from 323 to 473 K, at 50K intervals. For each temperature scenario, we have studied a range of pressure gradients and contact angles (see Table 1 in the Methods Section). Rock wettability shows a large variability from water-wet to CO$_2$-wet^19^. Clean mineral surfaces, such as quartz, calcite, feldspar, and mica are water-wet (CO$_2$ contact angle ranges between 120$^{\circ }$ and 180$^{\circ }$) due to their hydrophilic character. In subsurface systems at reservoir conditions, the presence of organic matter is very likely. Typically, these surfaces were aged in crude oil or coal and are CO$_2$-wet or intermediate-wet, with CO$_2$ contact angles ranging from 10$^{\circ }$ to 110$^{\circ }$^19^. In the following, we define the contact angle with respect to the injected fluid (scCO$_2$), which is the complementary of the angles normally defined in the literature^4,12,19^, see Table 2.

**Table 1:** Simulation scenarios. Set of parameters used in the two-phase simulations, for each temperature scenario of interest.

	Contact	Interfacial	Viscosity (Pa.s)	Viscosity (Pa.s)	Pressure
Scenario	Angle ($^\circ$)	Tension		Supercritical	Gradient
	Range	(mN/m)	Water	$C0_2$	(Pa/m)
323 K	20-110	$\sim$27	54.65 $\times {10}^{-5}$	3.26 $\times {10}^{-5}$	1 $\times {10}^4$ -1 $\times {10}^7$
373 K	20-110	$\sim$40	28.158 $\times {10}^{-5}$	2.22 $\times {10}^{-5}$	1 $\times {10}^4$ -1 $\times {10}^7$
423 K	20-110	$\sim$42	18.261 $\times {10}^{-5}$	2.23 $\times {10}^{-5}$	1 $\times {10}^4$ -1 $\times {10}^7$
473 K	20-110	$\sim$42	13.459 $\times {10}^{-5}$	2.38 $\times {10}^{-5}$	1 $\times {10}^4$ -1 $\times {10}^7$

**Table 2:** Representative parameter values. Parameters used to estimate the contributions to the pressure difference on a two-phase simulation representative of our study.

Parameter	Symbol	Value
Temperature (K$^\circ$)	T	373
Absolute pressure (Pa)	P	1 ×10^6^
Pressure gradient (Pa/m)	$\Delta P$	1 ×10^6^
Water viscosity (Pa.s)	$\eta _2$	28 ×10^−5^
Supercritical C0_2_ viscosity (Pa.s)	$\eta _1$	2.22 ×10^−5^
Water density (Kg/m$^3$)	$\rho _2$	960
Supercritical C0_2_ density (Kg/m$^3$)	$\rho _1$	145
Interfacial tension (N/m)	$\sigma$	40 ×10^−3^
Gravitational acceleration (m/s$^2$)	g	9.81

Parameter	Symbol	Value
Average capillary diameter (m)	D	20 ×10^−6^
Average capillary length (m)	L	2.25 ×10^−6^
$\sin \beta$	$\sin \beta$	0.5
Interface position approx (m)	*x* = L/2	1.125 ×10^−6^
Interface speed approx (m/s)	$\dot{x}$	5 ×10^−3^
Interface acceleration approx (m/s$^2$)	$\ddot{x}$	5 ×10^−3^

Another parameter with large variability is temperature. Low-temperature reservoirs range from 293 K to 363 K, intermediate-temperature reservoirs from 363 to 423 K, and high-temperature reservoirs from 423 to 573 K^30^. In our studies, we have considered temperatures ranging from 323 to 473 K, see Table 1, representing increasingly deeper injection points in reservoirs, assumed a fixed absolute pressure of 10 MPa, and set a range of pressure gradients between 1$\times 10^4$ and 1$\times 10^7$ Pa/m for driving the flow. Parameters of viscosity for the resident and injected fluids as well as the interfacial tension between the fluids, necessary to perform two-phase flow simulations, were extracted from the literature^31–33^. At the temperatures considered in this work, we have applied the correlating equation for the viscosity of water as extracted by Huber et al.^31^ in terms of the product of a temperature-dependent zero-density limit term, and a residual viscosity term that depends of both temperature and density as its value increases. The density of water at each value of temperature and pressure was calculated according to Wagner et al.^34^. We have computed the viscosity of CO$_2$ under supercritical conditions of pressure and temperature following the correlation extracted by Heidaryan et al.^32^. Finally, we have deduced the interfacial tension between supercritical CO$_2$ and water at the reservoir pressure for the various temperature scenarios from the work of Bachu and Bennion^33^ as provided in Table 1.

**Figure 2:**
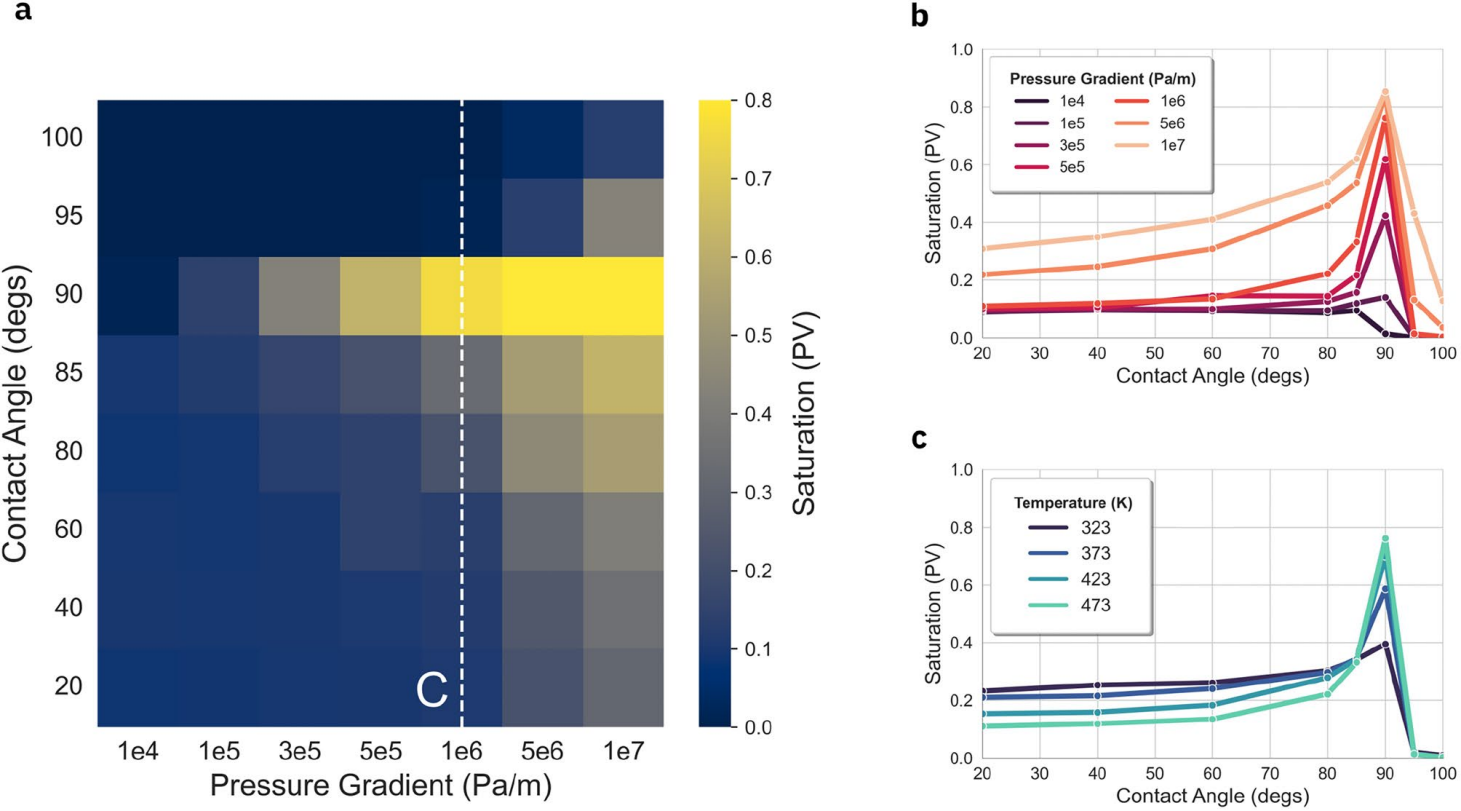
CO$_2$ saturation as function of pressure and contact angle. (**a**) Distributional map of the maximum CO$_2$ saturation as a function of applied pressure gradient and contact angle, respectively, at a temperature of 473 K. (**b**) Maximum CO$_2$ saturation as function of contact angle for representative pressure gradients at a temperature of 473 K. (**c**) CO$_2$ saturation along *C* cutline at a pressure gradient of 1$\times 10^6$ Pa/m for representative temperatures.

Figure 2a,b show the maximum scCO$_2$ saturation curves at T = 473 K plotted as function of fluid interface contact angle. The maximum scCO$_2$ saturation was extracted from the saturation as a function of time, as shown in Fig. S10a. Each point in Fig. 2b represents the maximum value of CO$_2$ saturation from the average across 150 simulations (set of 50 simplified capillary networks with flow along X, Y and Z axes) per set of injection conditions. Contact angles between 20$^{\circ }$ and 80$^{\circ }$ represent CO$_2$-wet regime where higher saturation by the injected fluid would be expected as capillary pressure favors the displacement of the resident fluid, particularly when, as per the calculations in Table 3, viscous forces remain lower than the externally applied. We observe that for pressure gradients below 1$\times 10^5$ Pa/m, the maximum CO$_2$ saturation is around 15$\%$. This saturation value is almost constant for contact angles ranging from 20$^{\circ }$ to 80$^{\circ }$ (CO$_2$ wet range), with a slight increase around 85$^{\circ }$ for pressure gradient 1$\times 10^5$ Pa/m. For larger contact angles (water-wet), the CO$_2$ saturation diminishes to zero. We note that the CO$_2$ saturation behaviour is a result of the collective interactions between all the pores in the capillary network and reflects the competing interplay between the externally applied pressure gradient and the balance of capillary pressures and viscous forces within each capillary of the network, see Eq. 3. While the observed saturation behaviour is surprising and might appear counter-intuitive, it emerges from the complex interplay of capillaries in the network and indicates that pore scale analysis may reveal unexpected phenomena at larger scales. For externally applied pressure gradients above 1$\times 10^5$ Pa/m, we observe in Fig. 2a,b a strong dependence of CO$_2$ saturation on contact angle. In particular, saturation of CO$_2$ increases monotonically as function of contact angle within the intermediate-wetting regime^35^ until reaching a peak around 90$^{\circ }$. Beyond that angle, CO$_2$ saturation rapidly decreases with the contact angle as the surface wettability turns to water wet.

**Table 3:** Contributions to the pressure gradient per capillary. Contributions to the pressure difference at both ends of each capillary on a two-phase flow simulation based on typical parameter values.

Pressure difference: outlet–inlet per capillary (N/m2)					
Externally applied	Capillary	Viscous	Hydrostatic	Kinetic	Inertia
$\Delta P \cdot L$	$-\frac{4\sigma }{D}cos\theta$	$\frac{32}{D^{2}}(\eta _{2}(L - x) + \eta _{1}x)\dot{x}$	$g(\rho _{2}(L - x) + \rho _{1}x)sin\beta$	$\dot{x}^{2}(\rho _2 + \rho _{1})$	$\ddot{x}(\rho _{2}(L-x) + \rho _{1}x)$
2.25	$-$8000 cos$\theta$	0.135	6.1$\times$10$^{-3}$	0.0275	6.2$\times$10$^{-6}$

In the regime of pressure gradients below 1$\times 10^5$ Pa/m, fluid flow is mostly driven by capillary pressure. Applying the second term of the right hand side of Eq. 3 to the range of capillary diameters shown in Fig. 1b, we estimate the capillary pressure to be of the order of 4 kPa for contact angles between 20$^\circ$ and 80$^\circ$, the same order of magnitude as the simulated pressure distributions on capillary nodes shown in Fig. S9a. When the fluid-solid interface contact angle $\theta$ approaches 90$^\circ$ degrees, capillary pressure drops to zero, see Eq. 3. In this pressure regime, CO$_2$ saturation diminishes as the driving force is not strong enough for sustaining fluid flow. At low externally applied pressure gradients, the magnitude of the pressure within the capillary network is nearly negligible, see Fig. S9b. The relatively low CO$_2$ saturation levels observed for some contact angles are likely due to the network complexity and the remaining viscous forces, with capillaries being plugged by interfaces of water/CO$_2$, where the capillary pressure counteracts fluid flow.

The higher CO$_2$ saturation seen in the regime of pressure gradients above 1$\times 10^5$ Pa/m stems from the fact that the pressure gradients induced by the external driving force are strong enough to overcome the counteracting capillary pressure on those capillaries that had become plugged under conditions of low contact angles, and thus enabling reaching significantly higher saturation values. Saturation values of 20$\%$ to 40$\%$ are observed for gradient pressure of 5$\times 10^6$ and 1$\times 10^7$ Pa/m, respectively–see Fig. 2a. In Fig. S9a, we observe nodes with pressure of the same order of magnitude as the capillary pressure. Moreover, within the intermediate-wet regime, as the contact angle approaches 90$^\circ$, CO$_2$ saturation reaches a peak because capillary pressure drops to zero and fluid flow is driven primarily by the applied pressure gradient. Within this regime, the saturation values of CO$_2$ decrease rapidly with contact angles larger than 90$^\circ$, because the imposed pressure gradient is no longer high enough to counteract capillary pressure.

Figure 2a maps the maximum achievable CO$_2$ saturation distribution with regards to applied pressure gradient and of CO$_2$-water-rock contact angle. The distinct region of sharp saturation increase around the 90$^\circ$ can be seen in Fig. 2b,c along the 2 cross-sectional line indicated as *C* in Fig. 2a. As a key result of our study, we obtain a maximum of 85.6% CO$_2$ saturation for a pressure gradient of $10\times 10^7$ Pa/m and $T=473\,\text {K}$ using $90^\circ$ contact angle, for the representative sandstone sample. We also observe in Fig. 2b, that the saturation increases with the applied pressure until it reaches a plateau. Variability of the saturation as a function of temperature is generally small as can be seen in Fig. 2c. The increased saturation following deeper permeation of the rock space by scCO$_2$ during injection within the intermediate-wet regime is consistent with a lower capillary pressure^12^. In the following, we will analyze the injection conditions for CO$_2$ drainage displacement in view of injection security and process efficiency.

### Optimization of CO$_2$ drainage displacement conditions

**Figure 3:**
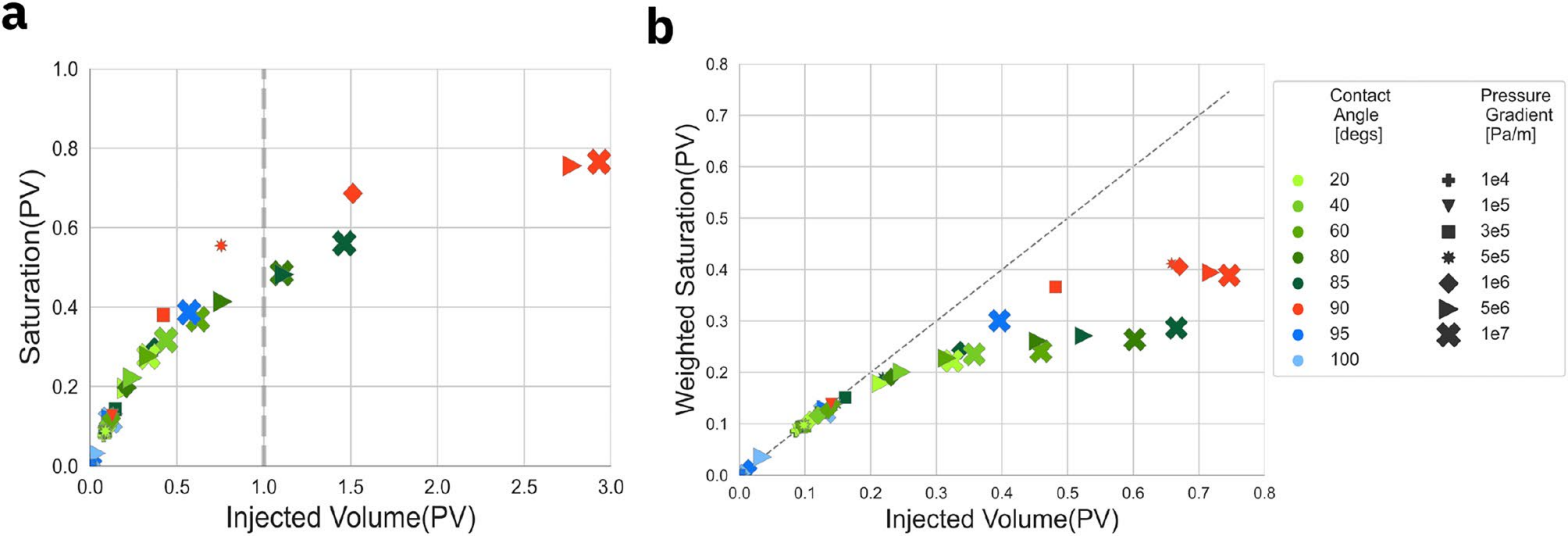
Efficiency and security of CO$_2$ drainage saturation and retention towards capillary trapping potential. Green color shades represent the CO$_2$-wet regime, blue shades correspond to the water-wet regime, and red shades identify contact angles close to 90$^\circ$. The temperature is set to 473 K. Colors represent contact angle while symbols represent pressure gradient. Larger symbols represent higher pressure gradients. The legend is common to both plots. (**a**) CO$_2$ saturation at 90$\%$ of the maximum value as a function of the injected volume required to reach that value. Injected volume larger than 1 PV represents scCO$_2$ that is not stored within the sample. (**b**) Relation of maximum weighted CO$_2$ saturation (*wS*) to injected volume across simulated conditions, aimed at increasing saturation close to the diagonal for maximum injection utilization.

CO$_2$ injection security and costs are primary concerns when optimizing injection conditions. Injected fluid that is not trapped within the pore space will escape. This occurs when the injected volumes surpass one pore volume (PV). CO$_2$ storage security refers to the safe saturation potential capable of hindering leakage, avoiding CO$_2$ injected that is not trapped from reaching the outlet of the pore media. Fig. 3a sheds light on the relative amounts of retained *vs.* mobile scCO$_2$ within the sample by plotting the value of saturation as a function of the injected volume. Since the CO$_2$ saturation curve reaches a plateau at around 90$\%$ of the maximum value (see Fig. S10a and b), to maintain consistency between simulation conditions, this value of saturation was selected to determine the representative injected volume of each case analyzed. We observe in Fig. 3a that intermediate-wet conditions lead to the largest saturation values, especially around a contact angle of 90$^\circ$ and at higher pressures. However, this often requires injection of volumes larger than 1 PV. While the exact values depend on the injection conditions, the results suggest that intermediate-wet conditions may require lower injection pressures to enhance injection security. At lower temperatures, we observe a reduced level of saturation per injected volume, see Fig. S12, requiring larger injected volumes.

Operational efficiency of the process requires maximizing the volume of CO$_2$ retained while minimizing the volume of CO$_2$ injected, thus minimizing cost. The fraction of the injected CO$_2$ that passes through the pore space without being retained leads to the saturation curve of scCO$_2$ forming a plateau as a function of time, as in Fig. S10a. This effect can also be analyzed as function of injected volume, see Fig. S10b, for the same simulation conditions. For the purpose of our analysis, we define the variable *Weighted Saturation* (*wS*) as the CO$_2$ saturation (*S*) scaled by the ratio of saturation to injected volume (*IV*), that is, $wS = S\frac{S}{IV}$, with units of pore volumes of injected scCO$_2$ between 0 and 1. The peak value of *wS* is reached for a value of injected volume below 1, as any additional injection does not further increase the saturation level. In Fig. S10c, we plot the weighted saturation as a function of injected volume for a range of pressures at a contact angle of $85^\circ$, and Fig. S10d displays the weighted saturation for a range of contact angles at a fixed 5$\times 10^6$ Pa/m applied pressure gradient. The plot in Fig. 3b represents maximum weighted saturation relative to injected volume across all the simulated scenarios aimed at optimizing saturation close to maximum injection utilization. For contact angles around 90$^\circ$ and high applied pressures, we obtain the highest saturation values. However, lower applied pressures in the intermediate-wet regime like $90^\circ$ and $3 \times 10^5 - 5 \times 10^5$ Pa/m, might offer a better balance of safety and efficiency. Overall, higher temperatures seem to improve efficiency as well as safety, see Fig. S12 and Fig. S13.

In summary, we have developed a methodology to perform two-phase flow simulations in porous rock samples for exploring the drainage displacement of supercritical carbon dioxide in brine-flooded sandstone reservoirs under intermediate- and CO$_2$-wet conditions. We have analyzed a wide range of injection scenarios by varying contact angles, temperatures, and pressure gradients for identifying the best conditions for CO$_2$ storage potential through capillary trapping. Our CO$_2$ saturation results for contact angles between 20$^\circ$ and 40$^\circ$ are comparable with Krevor et al.^3^ for water-wet surfaces, where trapped saturation can reach up to 30% of the rock’s pore volume. For contact angles approaching the intermediate-wet regime around around 90$^\circ$, however, the CO$_2$ saturation efficiency increases sharply. For optimized conditions, we obtain drainage displacement saturation values as high as 80% of the pore volume. Our results suggest that a high saturation efficiency may be maintained even at lower pressure levels which would significantly reduce the risk of CO$_2$ leakage.

## Methods

### Simulation scenarios and parameters

Table 1 collects the parameters used in our simulations.

### Digital rock image processing

This work relies on high-resolution digital rock images of rock samples as the geometrical basis for the pore scale simulations and the study of rock properties and fluid infiltration and storage within sub-surface porous structures^24,36^. In our work, we have acquired and shared as open data a large dataset of three-dimensional images extracted from X-ray $\mu$CT scans^24,27^ of sandstone and carbonate rock samples. For this work, we run our simulation analysis on a Berea Sister Gray sandstone sample randomly selected among the available rocks. The reconstructed volume of the fully digitized rock sample obtained from the $\mu$CT measurements is usually cropped into cube-shaped volumes that are more computationally manageable while retaining statistically significant rock properties. In our simulations, we employed a $(2.25\,\text {mm})^3$ digitized sandstone rock sample scanned at a resolution of $2.25\,\mu \text {m/voxel}$. This resulted in a digital sample with $1000^3$ voxels that was determined as the Representative Elementary Volume (REV) and therefore sufficient to yield accurate rock property predictions like porosity and permeability^24^. More details can be found in the Supplementary Information Section S1.

### Capillary network extraction and centerlines representation

We use a graph-based, capillary network model (CNM) as an accurate representation of the rock porous geometry. Starting with the 3D binarized digital rock image, we apply a custom Dijkstra’s Minimum Path algorithm^37^, transforming the pore space into voxel-wide lines at the center of the pore channels, finding the most central path from inlet pore to outlet pore of each set of connected pore space voxels through a centrality-based cost function. Each section of the line is saved as a node in a graph and converted into a short cylinder with spatially varying radius to match the local geometry extracted from the microtomography. The resulting network of connected capillaries is an accurate representation of the rock’s pore space. As an example of this transformation, we show the small $100^3$ image with $(225\,\mu \text {m})^3$ volume and its corresponding CNM in Fig. S2 containing 4069 capillaries with a color-coded diameter scale. The capillary network of the sandstone sample analyzed in this paper displays a porosity of $33\%$ and results in a CNM with $2.7 \times 10^6$ capillaries. More details can be found in the Supplementary Information Section S2, and the full algorithm description can be found in^24^.

### Single-phase flow simulation

The fined-grained capillary network representation of the rock’s pore spatial distribution described in the previous section was employed to simulate both single and two-phase fluid flow with a high level of geometrical accuracy. We assume laminar flow and apply the equations relating pressure and flow within each capillary, followed by conservation of mass at each network node, to build a large system of coupled equations in sparse matrix form. The Hagen-Poiseuille Eq. (1) is applied when simulating single-phase stationary flow, that is,

1 $$\begin{aligned} Q_j=\frac{\pi R_j^4}{8 \mu L_j} \Delta P_j , \quad \quad \text {for every capillary } j \end{aligned}$$

where $R_j$ and $L_j$ denote, respectively, the radius and length of capillary *j*, $\mu$ is the viscosity of the fluid, and $Q_j$ and $\Delta P_j$ represent the flow rate and pressure difference across each capillary *j*. Combining Eq. (1) with the conservation of mass at each network node, Eq. (2),

2 $$\begin{aligned} \sum _j Q_{i,j} = 0, \quad \quad \text {for every node } i, \end{aligned}$$

results in a large but rather sparse system of equations to extract properties like pressure distribution or flow rate at each point in the network of microscopic capillaries, as well as bulk flow properties like permeability^24^. Fig. S3 displays the results of running single-phase, pressure-driven, Poiseuille flow simulations on the capillary network representation of a $1000^3$ voxel sandstone rock sample as input geometry. The absolute permeability for this REV was computed from these simulations as 105, 92 and 54 mD along the X-, Y- and Z-axis, respectively. Further description can be found in the Supplementary Information Section S3.

### Two-phase flow simulation

Two-phase flow simulations track the displacement in time of the fluid-fluid interface within each capillary of the high-resolution 3D geometric representation of the rock sample. We restrict the modeling to two incompressible fluids, under laminar, one-dimensional flow along the length of each capillary. Each fluid-fluid interface is assumed perpendicular to the flow direction, *i.e.* piston-like displacement. The pressure gradient between the two ends of each capillary is expressed as the sum of the gradients produced by various physical effects acting on the position *x*(*t*) of the effective interface, after removing all annulling interfaces, between fluids, that is,

3 $$\begin{aligned} {\Delta P}_j\ = \frac{8}{R_j^2}\left[ \mu _1 x_j + \mu _2 \left( L_j - x_j \right) \right] {\dot{x}}_j\ -\frac{2\sigma }{R_j} \cos {\theta } \quad \quad \text {for each capillary}\,j , \end{aligned}$$

where ${\Delta P}_j$ is the pressure difference across capillary *j*, with length $L_j$ and radius $R_j$. $\mu _i$ represents the viscosity of fluid *i* and $\sigma$ the interfacial tension between fluids, while $\theta$ describes the contact angle formed by both fluids and the matrix surface, defined with reference to the injected fluid as illustrated in Table 2. Finally, $x_j$ and ${\dot{x}}_j$ denote the position of the effective interface and its first derivative, respectively. The first term on the right-hand side refers to viscous forces, and second term refers to the capillary pressure contribution from the interfaces. This contribution cancels out in capillaries with an even number of (alternating) interfaces, and reverts to something similar to Eq. (1). Any odd number of interfaces can be represented by a single “effective” interface whose position is calculated as to preserve the relative saturation of each fluid.

In our current implementation, we retain the effect of viscous forces and first order capillary pressure, after higher order dynamical effects to the interface shape are dismissed. Table 3 summarizes the expressions and estimated values of these physical effects, based on the assumptions in Table 2. The average capillary length (i.e., one voxel) and diameter are representative of the distribution shown in Fig. 1b. Under these assumptions, the contributions from hydrostatic, kinetic and inertial forces appear at least one order of magnitude smaller than the least significant of the other three contributions, hence the choice of terms kept in Eq. (3). Together with mass conservation at the nodes, Eq. (3) forms a system of differential-algebraic equations (DAE) representing the interface dynamics over time. Tracking of the fluid interfaces across the network of capillaries then proceeds in alternating sequences of *free evolution* and *jumps*. Free evolution refer to the time interval in which the interfaces progress along the same capillary and the overall number of interfaces within all capillaries in the network remain constant. Jump steps occur when any interface reaches a node, which means that the interface is ready to leave its current capillary and enter one or more new capillaries. In this step, free evolution pauses, the interfaces are redistributed throughout the network, and the system of differential algebraic equations representing the network dynamics is rewritten to account for the changes in interface locations. The simulator computes the velocity of the fluid flow through all capillaries as a function of time, which is integrated to determine the position of the fluid-fluid interface within each capillary at each time step, from which to deduce the level of saturation of each fluid phase in the rock pore space. More details on the formulation and implementation can be found in Section S4 of the Supplementary Information.

### Simplified capillary network representation

Unlike single phase flow that can run within minutes even on the high-resolution capillary network representation of a REV-sized rock sample with millions of nodes and links, the two-phase dynamic simulations become unfeasible even on large computing resources. To overcome this limitation, we employ a smaller capillary network yet representative of the original rock full centerlines representation to run two-phase simulations and extract flow properties. A custom algorithm is used to construct simplified 2D and 3D capillary networks capable of preserving geometrical properties of the rock morphology relevant to fluid flow such as : (i) porosity; (ii) capillary diameter distribution; and (iii) node coordination number distribution^38^. The method iterates over the following steps until the porosity of the synthesized network is within a small increment from the original. First it selects a coordination number for each node applying the probability distribution of the original network and adjusts the number of capillaries connected to each node to match the assigned number. Then capillary diameters are assigned by randomly choosing from the diameter probability distribution of the original capillary network. The final step in each iteration involves calculating the porosity of the simplified network by dividing the capillary volume by the sample volume, and informing the adjustments in number of nodes and capillaries of the following iteration based on the comparison against the porosity of the original rock sample. Examples of probability distributions of capillary diameters and coordination numbers, and 2D and 3D ssCN in regular and random configurations can be seen in Fig. S6 and S7, respectively.

Alternative methods propose generating stochastic network models extracted from alternative network modeling approaches^38–40^. Our capillary network representation of the rock geometry has shown superior feature resolution at the microscopic scale and more accurate estimation of plug-scale rock properties^24^. Consequently, stochastic capillary networks built to closely match the morphology of the original rock will more accurately represent the geometry when using a capillary network representation of the original sample. More details on the algorithm to synthesize networks can be found in Section S5 of the Supplementary Information.

### Simulation toolkit for scientific discovery (ST4SD)

In our study we scanned through 4 different temperature scenarios, and per scenario, we studied about 8 fluid-rock interface contact angle values and no less than 8 different driving pressure gradient cases per angle, totaling in 256 different injection conditions to be simulated. Per case simulated, flow simulations of a full data-set of 50 simplified capillary networks, assuming driving pressure along all three axes, that is, 150 executions per each of the 256 cases considered, requiring proper parsing and aggregation of nearly $4 \times 10^4$ simulations. In this work, we employed the Simulation Toolkit for Scientific Discovery (ST4SD) ^41^ to automate the execution of long simulation campaigns with several chained steps. The use of such workflow scheduler ensures the reproducibility of our results and enable efficiency gains by optimising the use of computing resources. Fig. 1 illustrates the conceptual workflow and in Fig. S11, we show the sequence of steps executed as an ST4SD experiment. A CNM representation of a rock sample is used as input to the ST4SD routine. In this workflow we generate tens to hundreds of simplified capillary networks that meaningfully represent the properties of the original rock sample network model (see Section *Simplified capillary network representation*). Each simplified capillary network is then used as geometrical input to parallel flow simulations that will estimate relevant physical properties in each representative system. Finally, the individual results from each simplified network are aggregated and combined into a single estimate that applies to the original network. See Supplementary Information Section S8 for more details on this workflow.

### Scalability and applicability

The size of the original sandstone rock sample was selected as the minimum representative elementary volume (REV) that can yield accurate rock property predictions of porosity and permeability of the larger rock plug^24^. Extrapolation of these microscopic results to a reservoir scale can, in principle, be achieved by applying the relative permeability and capillary pressure curves as input parameters of a macroscopic continuum model. Following Kløv et al.^42^, the rock facies are discretized in regular grids in horizontal and vertical directions, and each rock type in the grid characterized by an average absolute permeability and porosity and a set of average rock curves (relative permeability and capillary pressure) derived from the microscopic capillary network model.

In this work we have studied the injection of CO$_2$ into subsurface porous formations during the drainage phase of the storage process. Once the externally applied pressure is removed, the previously displaced brine flows back towards the pore space during the post-drainage imbibition phase. To study this phase and quantify the final volume of CO$_2$ trapped requires implementing extensions to the code to enable three-phase flow and the inclusion of dynamic boundary conditions to simulate a water-alternate-gas injection scenario. We have open-sourced the digital rock and flow simulation program code to enable a community-driven reuse and extension of its capabilities.

### Supplementary Information

Supplementary Information 1.

Supplementary Information 2.

## Data Availability

Simulation data in the form of saturation over time is available as nn information (*Saturation-Data-Berea.xlsx*). Microtomography datasets containing grayscale and binary rock image data are available at the Digital Rocks Portal (https://dx.doi.org/10.17612/f4h1-w124) for sandstone samples and on Figshare (10.25452/figshare.plus.21375565.v6) for sandstone and carbonate samples.

## Code availability

The source code used to extract the CNM representation from the rock $\mu$CT image is available at https://github.com/IBM/flowdiscovery-digital-rock. Additional algorithms used for processing and segmenting the raw grayscale images, are available as Python code at https://github.com/IBM/microCT-Dataset. The code used to run single-phase and two-phase flow simulations on CNM representations of the rock samples is available at https://github.com/IBM/flowdiscovery-simulator. The code employed in the automation of the scientific workflows allowing the execution of ST4SD ^41^ experiments is available at https://github.com/st4sd/flow-simulator-experiment.

## References

[1] Metz B, Davidson O, De Coninck H, Loos M, Meyer L (2005). IPCC Special Report on Carbon Dioxide Capture and Storage.

[2] Kramer D (2020). Negative carbon dioxide emissions. Phys. Today.

[3] Krevor S (2015). Capillary trapping for geologic carbon dioxide storage–from pore scale physics to field scale implications. Int. J. Greenhouse Gas Control.

[4] Chiquet P, Broseta D, Thibeau S (2007). Wettability alteration of caprock minerals by carbon dioxide. Geofluids.

[5] Saraji S, Goual L, Piri M, Plancher H (2013). Wettability of supercritical carbon dioxide/water/quartz systems: Simultaneous measurement of contact angle and interfacial tension at reservoir conditions. Langmuir.

[6] Al-Khdheeawi EA, Vialle S, Barifcani A, Sarmadivaleh M, Iglauer S (2017). Influence of co2-wettability on co2 migration and trapping capacity in deep saline aquifers. Greenh. Gases: Sci. Technol..

[7] Al-Menhali AS, Krevor S (2016). Capillary trapping of CO$$_2$$ in oil reservoirs: Observations in a mixed-wet carbonate rock. Environ. Sci. Technol..

[8] Krevor SCM, Pini R, Zuo L, Benson SM (2012). Relative permeability and trapping of CO$$_2$$ and water in sandstone rocks at reservoir conditions. Water Resour. Res..

[9] Shi J-Q, Xue Z, Durucan S (2011). Supercritical CO$$_2$$ core flooding and imbibition in berea sandstone–CT imaging and numerical simulation. Energy Procedia.

[10] Bennion, D. B. & Bachu, S. Drainage and imbibition CO$$_2$$/brine relative permeability curves at reservoir conditions for carbonate formations. In All Days (SPE, 2010). https://doi.org/10.2118/134028-ms.

[11] Bennion DB, Bachu S (2008). Drainage and imbibition relative permeability relationships for supercritical CO$$_2$$/brine and H$$_2$$S/brine systems in intergranular sandstone, carbonate, shale, and anhydrite rocks. SPE Reserv. Eval. Eng..

[12] Hu R, Wan J, Kim Y, Tokunaga TK (2017). Wettability effects on supercritical CO$$_2$$-brine immiscible displacement during drainage: Pore-scale observation and 3D simulation. Int. J. Greenh. Gas Control.

[13] Gooya R (2019). Unstable, super critical CO$$_2$$-water displacement in fine grained porous media under geologic carbon sequestration conditions. Sci. Rep..

[14] Kalam S (2020). Carbon dioxide sequestration in underground formations: review of experimental, modeling, and field studies. J. Pet. Explor. Prod. Technol..

[15] Hinton EM, Woods AW (2021). Capillary trapping in a vertically heterogeneous porous layer. J. Fluid Mech..

[16] Guo R (2022). Role of heterogeneous surface wettability on dynamic immiscible displacement, capillary pressure, and relative permeability in a CO$$_2$$-water-rock system. Adv. Water Resour..

[17] Alhosani A (2020). Pore-scale mechanisms of CO$$_2$$ storage in oilfields. Sci. Rep..

[18] Plug W-J, Bruining J (2007). Capillary pressure for the sand-co2-water system under various pressure conditions. Application to co2 sequestration. Adv. Water Resour..

[19] Iglauer S (2017). CO$$_2$$-water-rock wettability: Variability, influencing factors, and implications for CO$$_2$$ geostorage. Acc. Chem. Res..

[20] Huang H, Meakin P, Liu M (2005). Computer simulation of two-phase immiscible fluid motion in unsaturated complex fractures using a volume of fluid method. Water Resour. Res..

[21] Pan C, Hilpert M, Miller CT (2004). Lattice-boltzmann simulation of two-phase flow in porous media. Water Resour. Res..

[22] Valvatne PH, Blunt MJ (2004). Predictive pore-scale modeling of two-phase flow in mixed wet media. Water Resour. Res..

[23] Raeini AQ, Bijeljic B, Blunt MJ (2017). Generalized network modeling: Network extraction as a coarse-scale discretization of the void space of porous media. Phys. Rev. E.

[24] Neumann RF (2021). High accuracy capillary network representation in digital rock reveals permeability scaling functions. Sci. Rep..

[25] Tirapu-Azpiroz, J. *et al.* Cloud-based pore-scale simulator for studying carbon dioxide flow in digital rocks. In *Proceedings of the 16th Greenhouse Gas Control Technologies Conference* (GHGT-16) (2022). https://ssrn.com/abstract=4276744.

[26] Lucas-Oliveira, E. *et al.* Micro-computed tomography of sandstone rocks: Raw, filtered and segmented datasets. Data Brief. **41** (2022). 10.1016/j.dib.2022.107893 PMC8844843 35198674

[27] Esteves Ferreira M (2023). Full scale, microscopically resolved tomographies of sandstone and carbonate rocks augmented by experimental porosity and permeability values. Sci. Data.

[28] Neumann, R., Andreeta, M. & Lucas-Oliveira, E. 11 sandstones: Raw, filtered and segmented data. http://www.digitalrocksportal.org/projects/317 (2020). 10.1016/j.dib.2022.107893 PMC8844843 35198674

[29] Ferreira ME (2023). Full scale, microscopically resolved tomographies of sandstone and carbonate rocks augmented by experimental porosity and permeability values. Figshare.

[30] Zarrouk, S. J. & McLean, K. Chapter 2 - geothermal systems. In Zarrouk, S. J. & McLean, K. (eds.) Geothermal Well Test Analysis, 13–38 (Academic Press, 2019). https://www.sciencedirect.com/science/article/pii/B9780128149461000025.

[31] Huber ML, Perkins RA, Laesecke A, Friend DG (2009). New international formulation for the viscosity of H$$_2$$O. J. Phys. Chem. Ref. Data.

[32] Heidaryan E, Hatami T, Rahimi M, Moghadasi J (2011). Viscosity of pure carbon dioxide at supercritical region: Measurement and correlation approach. J. Supercrit. Fluids.

[33] Bachu S, Bennion DB (2009). Interfacial tension between CO$$_2$$, freshwater, and brine in the range of pressure from (2 to 27) MPa, temperature from (20 to 125)$$^{\circ }\text{C}$$, and water salinity from (0 to 334 000) mg$$\cdot$$L$$^{-1}$$. J. Chem. Eng. Data.

[34] Wagner W, Pruß A (2002). The IAPWS formulation 1995 for the thermodynamic properties of ordinary water substance for general and scientific use. J. Phys. Chem. Ref. Data.

[35] Iglauer S, Pentland CH, Busch A (2015). Co2 wettability of seal and reservoir rocks and the implications for carbon geo-sequestration. Water Resour. Res..

[36] Andrä H (2013). Digital rock physics benchmarks-part ii: Computing effective properties. Comput. Geosci..

[37] Dijkstra EW (1959). A note on two problems in connexion with graphs. Numer. Math..

[38] Raoof A, Hassanizadeh SM (2009). A new method for generating pore-network models of porous media. Transp. Porous Media.

[39] Idowu, N. A. Pore-scale modeling: Stochastic network generation and Modeling of rate effects in waterflooding. Ph.D. thesis, Imperial College London (2009).

[40] Sok RM (2002). Direct and stochastic generation of network models from tomographic images; effect of topology on residual saturations. Transp. Porous Media.

[41] Johnston, M. A., Vassiliadis, V., Pomponio, A. & Pyzer-Knapp, E. *Simulation Toolkit for Scientific Discovery* (2022). https://github.com/st4sd/.

[42] Kløv, T. *et al.* Pore-to-Field Scale Modeling of WAG. vol. All Days of SPE Annual Technical Conference and Exhibition, SPE–84549–MS (2003). https://doi.org/10.2118/84549-MS.

