# Supplementary material for: Enhanced carbon dioxide drainage observed in digital rock under intermediate wetting conditions
Source: Sci Rep. 2024 Jul 9;14:15852. doi: 10.1038/s41598-024-65920-6 (PMC11233577; doi:10.1038/s41598-024-65920-6)
Supplement: Supplementary file 1 — Supplementary Information 1. [file 41598_2024_65920_MOESM1_ESM.pdf]

Supplementary Information

Enhanced CO2 drainage observed in digital rock under intermediate wetting conditions

Jaione Tirapu Azpiroz[1], Ronaldo Giro[2], Rodrigo Neumann Barros Ferreira[1], Marcio Nogueira Pereira da Silva[1], Manuela Fernandes Blanco Rodriguez[1], Adolfo E. Correa Lopez[1], David A. Lazo Vasquez[3], Matheus Esteves Ferreira[1], Mariana Del Grande[1], Ademir Ferreira Da Silva[1], Mathias B. Steiner[1]

[1]*IBM Research, Av. República do Chile, 330, CEP 20031-170, Rio de Janeiro, RJ, Brazil.*
[2]*IBM Research, Rd J Fco Aguirre Proenca Km 9 Sp101, Hortolandia, Sao Paulo, 13186-900, SP, Brazil.*
[3]*IBM Research, Rua Tutóia, 1157, São Paulo, Sao Paulo, 04007-005, SP, Brazil.*

S1 Digital Rock Image processing

We used a large dataset of three-dimensional images extracted from X-ray computed micro-tomography (μCT) scans of sandstone and carbonate rock samples at a resolution of $2.25\ \mu$m/voxel.[24,27] The μCT system scans cylindrical rock plugs, as seen in Fig. S1a. A 26 mm-long and 10 mm-wide rock plug suitable for our system (Skyscan 1272, Bruker), was imaged as two-dimensional projections of the pore space. The three-dimensional digital rock image is reconstructed out of these two-dimensional projections using the built-in Bruker software (NRecon, version 1.7.0.4, with the Reconstruction engine InstaRecon, version 2.0.2.6). The reconstructed volume of the fully digitized rock sample obtained from the μCT measurements is usually cropped into smaller volumes that are more computationally manageable, while retaining statistically significant rock properties. For a set of sandstone rock samples scanned at a resolution of $2.25\ \mu$m/voxel, it was observed that a Representative Elementary Volume (REV) of about 1000^3 voxels was sufficient to yield accurate rock property predictions like porosity and permeability[24]. Additional image processing methods are applied to the digitized rock following the procedures described in references[24,43] to equalize contrast differences due to mineralogical compositions across images. To fully eliminate image and measurement artifacts resulting in very bright spots in the image, all voxels above a 99.8% threshold in the grayscale cumulative histogram have been removed and the remaining grayscale levels have been remapped to span the full [0, 255] scale. To reduce noise, a 3D non-local means filter[44,45] was applied, which is available in Fiji[46], using a smoothing factor of 1 and automatically estimated sigma[47] parameters.

Finally, several thresholding algorithms have been implemented to segment the grayscale images and separate the pore space (darker) from the rock matrix (lighter), depending on the rock

type. For instance, using a threshold level calculated by the IsoData method[48] was sufficient to accurately segment the sandstone grayscale images into solid (white) and void (black) space leading to binary images[24], but a 3-level multilevel Otsu method[49] was needed to properly group sub-porous regions, with little expected flow, with the mineral matrix for carbonate samples[27]. Fig. S1b displays an example of this segmentation process as applied to a $225\,\mu$m $\times$ $225\,\mu$m cross-section of a carbonate rock tomography, where the grayscale image on the left is segmented into the binary image on the right. The Enhanced Hoshen-Kopelman algorithm[50] is used to locate all pore clusters, determine the pore volume fraction, and eliminate the pore clusters that are not connected to the percolating pore network. The connected pore space is then taken as the true measure of porosity.

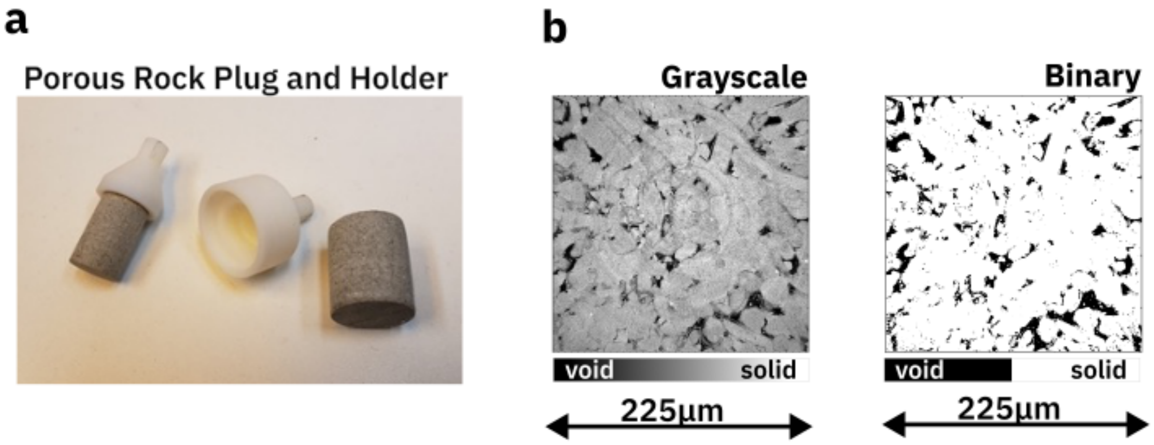

Supplementary Figure S1: **Digital rock image processing.** a) Examples of porous rock plugs and their plastic sample holders. b) Tomography cross section with dimensions $225\,\mu$m $\times$ $225\,\mu$m in grayscale (left) and its corresponding binarized image (right), scanned at a resolution of $2.25\,\mu$m/voxel.

S2 Capillary Network extraction and centerlines representation

The CNM representation is based on the Centerline extraction algorithm[24]. A centerline is a thin, one-dimensional object that captures a 3D object's main symmetry axes, summarizing its main shape into a set of curves[51,52]. Starting from the 3D binary image, our network extraction algorithm transforms the pore space into voxel-wide lines at the center of the pore channels, finding the most central paths from inlet pores to outlet pores through a centrality-based cost function inspired by the Dijkstra's Minimum Path algorithm [37]. The resulting graph is interpreted as a cylindrical capillary (a short cylinder), one voxel long, but whose diameter is the distance to the closest solid voxel. The full algorithm description can be found in Neumann *et al.*[24].

The digital rock image in Fig. S2a has dimension of $(225\,\mu\mathrm{m})^3$ and was digitized in 8-bit grayscale using one million (100^3) voxels, each representing a physical region of $(2.25\,\mu\mathrm{m})^3$. After

segmentation, about 26% of the voxels in the resulting binary image of Fig. S2b, are identified as pore space, 64.5% of which touch the rock surface. After undergoing the extraction procedure, the capillary network model of the sample provides an accurate representation of the rock porous geometry as seen in Fig. S2c. This CNM representation contains 4069 capillaries with a color-coded diameter scale. The capillary network of the Berea sandstone sample analyzed in this paper, of size equalto 1000^3 voxels, displays a porosity of 33% and results in a CNM with 2.7×10^6 capillaries.

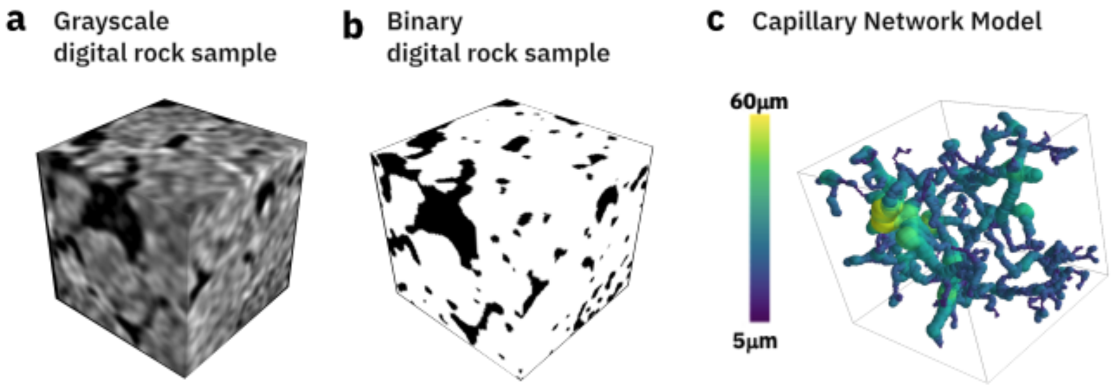

Supplementary Figure S2: **Capillary Network extraction**. 3D grayscale digital rock image (a) and 3D binary image (b) of a Berea sandstone sample with 100^3 voxels. Capillary network representation (c) of the sample mapping the rock pore geometry with a color-coded diameter

S3 Single-phase flow simulation

The fined-grained capillary network representation of the rock's pore space described in section S2 was employed to simulate both single and two-phase fluid flow with a high level of geometrical accuracy. We assume laminar flow and apply equations relating pressure and flow rate within each capillary, followed by conservation of mass at each node, to build a large system of coupled equations in sparse matrix form. The system solution contains local properties such as the distribution of pressure and flow rate at each point in the network, as well as global flow properties like permeability[24].

Fig. S3 displays the results of single-phase, pressure-driven flow simulations using the capillary network representation of a 1000^3 voxel sandstone rock sample as input geometry. A pressure gradient of 10 kPa/m between opposite sides along one axis is applied to drive the flow of a simple fluid with density 1000 kg/m^3 and dynamic viscosity 1.002 mPa.s, representing water at 1 atm, through the capillary network. Fig S3 is showing the induced pressure field inside the 3D sandstone sample of dimensions 2.25 mm $\times$ 2.25 mm $\times$ 2.25 mm. Due to the sparse nature of all matrices involved, single-phase flow simulations are very efficient and can be simulated within minutes, depending on its connected pore structure and the direction of the flow. The absolute permeability

for this REV was computed to be 105, 92 and 54 mD along the X-, Y- and Z-axis, respectively.

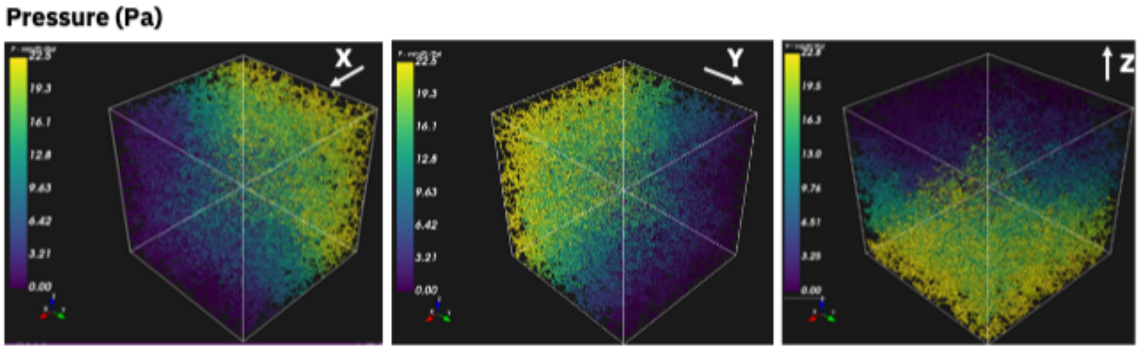

Supplementary Figure S3: **Single-phase fluid flow simulation**. Simulated pressure fields inside a capillary network induced by single-phase flow of a a simple fluid representing water at 1 atm . The flow was driven by an external 10 kPa/m pressure gradient imposed across the (a) X-axis (b) Y-axis and (c) Z-axis.

S4 Two-phase flow simulations

Two-phase flow simulations track the displacement in time of the fluid interface within each capillary of the CNM. We restrict the modeling to incompressible fluids under laminar, one-dimensional flow along the length of each capillary. Each fluid-fluid interface is assumed perpendicular to the flow direction, *i.e.* piston-like displacement. The pressure difference between the ends of each capillary is expressed in Eq. 3 as the sum of various physical effects, some depending on the position $x(t)$ of the fluid interface. In addition to the externally applied pressure gradient, viscous forces and capillary pressure, the physical equations of two-phase flow in capillaries may also include hydrostatic, kinetic, and inertia contributions. Under the assumptions of Table 2, the contributions from hydrostatic, kinetic and inertial forces appear at least one order of magnitude smaller than the least significant of the other three contributions, hence the choice of terms kept in Eq. 3.

Tracking of the fluid interfaces across the network of capillaries proceeds in alternating sequences of *free evolution* and *jumps*. During free evolution, the effective interfaces move along their respective capillaries, but without leaving them, via integration of the DAE system. Fig. S4a displays a simple network of 6 capillaries, represented by cap_i, where $i = 1..6$, sharing a single central node labeled with the pressure P_7 at that point. This example illustrates the free evolution time interval until the fluid interface in cap_6 reaches the central node. Jump events occur when an interface reaches a node and leaves its current capillary to enter one or more neighbouring capillaries. In this step, free evolution pauses, the interfaces are redistributed throughout the network, and the system of DAE is rewritten to account for the changes in interface locations. As an example, in Fig. S4b, an interface that reached the end of one capillary (cap_6) is removed, and new interfaces are created at the entrances of the connected capillaries. After this rearrangement, free evolution resumes. Depending on the local pressure state, some capillaries may become plugged, that is, the

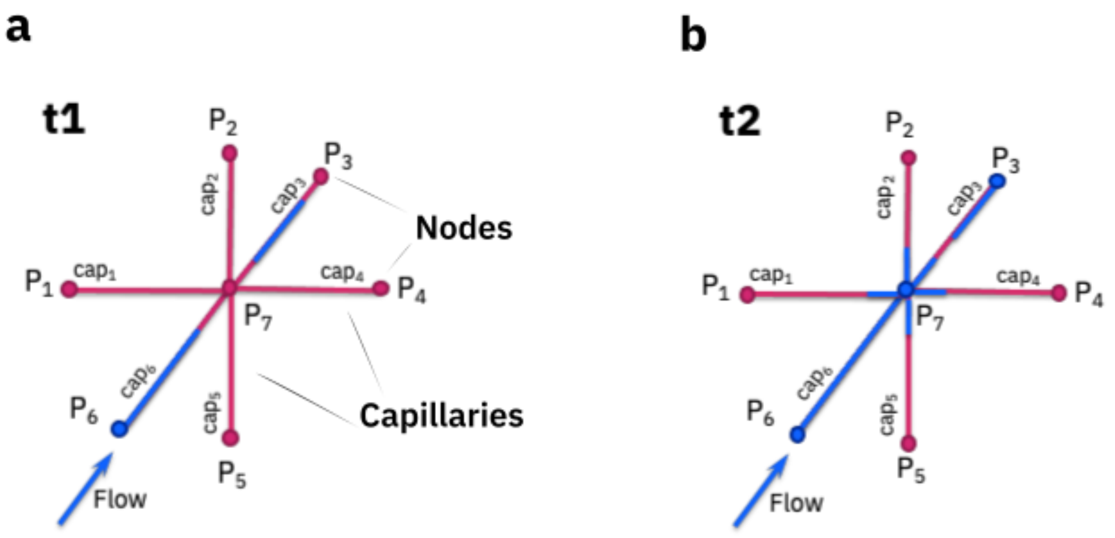

Supplementary Figure S4: **Fluid interface evolution in capillary network.** a) Time step where fluid interface progresses through the capillary before reaching a node, and b) time step after fluid interface in cap_6 reaches the central node and transitions to all connected capillaries.

pressure conditions may not favor flow and the interface becomes frozen at the location of nearest node. It is possible that events such as the merging of fluids, reversal of flow, or changes in the pressure conditions at a later time may start favoring flow again and lead to the capillary becoming unplugged. All possibilities are handled carefully during the redistribution of interfaces among the capillaries.

A more realistic example of the time evolution of a two-phase simulation is displayed in Fig. S5a and Fig. S5b, representing the injection of supercritical CO_2 over time as it pushes the resident water in a small portion of a Berea sandstone rock modeled as a network of connected capillaries. From the knowledge of the position of the fluid interface in all capillaries, we can compute the saturation as a function of time as displayed in Fig. S5c. Saturation of the rock sample by the injected fluid an important property in the study of CO_2 infiltration into the porous rock. Plotting the saturation as a function of the volume of CO_2 injected, as in Fig. S5d, provides a measure of the injection efficiency.

S5 Simplified capillary network representation

Unlike single-phase (stationary) flow that can be simulated within minutes, even on the network representation of a REV-sized rock sample with millions of nodes and links, dynamic simulations rapidly become unfeasible even on small sample sizes and large computing resources due to their time-dependent nature. To overcome this limitation, we employ sets of smaller capillary networks that remain representative of the original capillary network, to run two-phase simulations

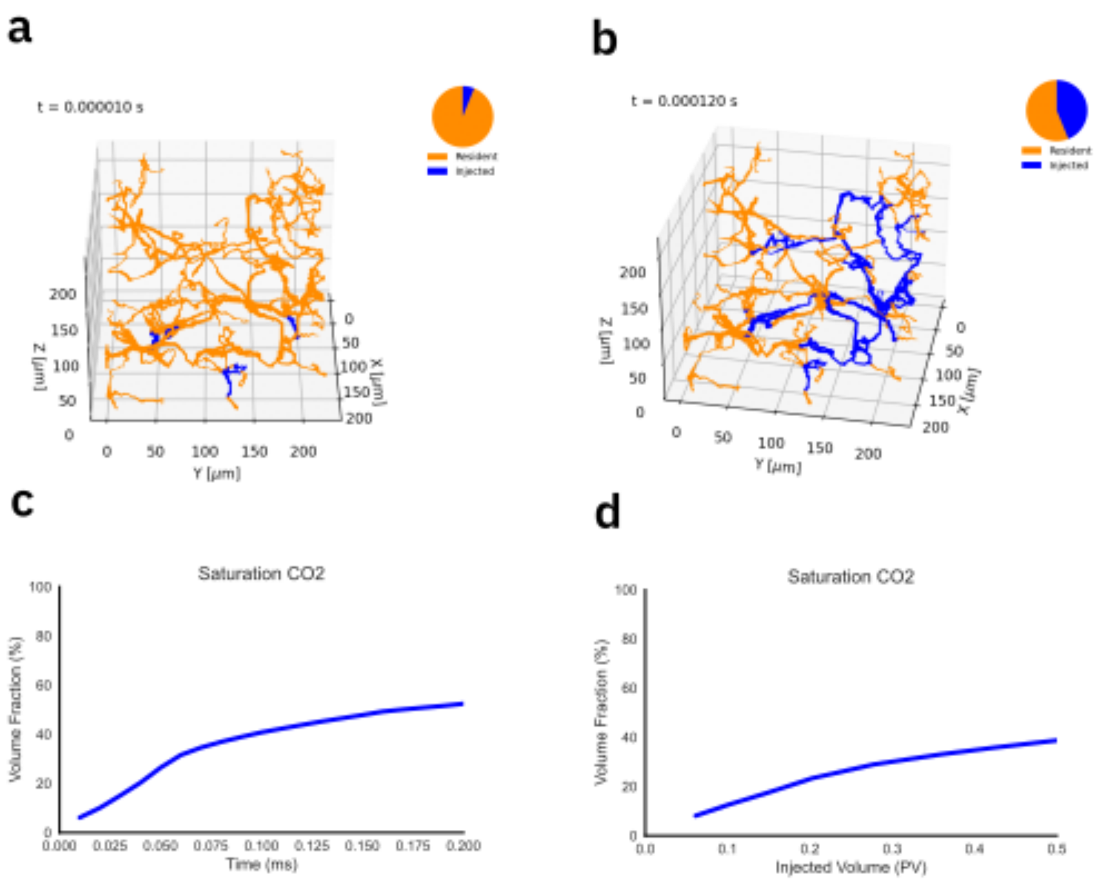

Supplementary Figure S5: **Two-phase fluid flow simulation**. Dynamics of two-phase fluid flow simulation at a) time t_1 and b) time t_2. Measure of scCO2 saturation into the porous rock as c) a function of time and d) a function of injected volume.

and extract flow properties. Previous works have demonstrated the importance of the geometric properties of porous media, in particular, the distributions of sizes and shapes of pores and throats, and also topology parameters such as connectivity and coordination number distribution[38]. Thus, in order to produce realistic predictions, our smaller and simplified capillary networks are required to accurately match the morphology of the original rock sample.

To create the simplified capillary network, we have developed a custom Python script based on the OpenPNM[53] framework capable of generating arbitrary 3D capillary networks in *regular* (cubic) and *random* configurations. A *regular* capillary network comprises a 3D cubic structure delimited by the sample size dimensions L_X, L_Y and L_Z, where capillaries are created parallel to the axes on a regular mesh, intersecting at regular intervals. The intersection between capillaries, referred to as a node, defines its coordination number. The coordination number is the number of

links associated to each node in the network. For example, in Supplementary Figure S4a the P7 point shows a coordination number of 6, while the rest of the nodes display a coordination number of just 1. One can create different cubic network configurations with coordination numbers from 6 to 26 by connecting faces, edges, corners, and their combination. In the *random* capillary network, a set of points (or nodes) are randomly distributed in 3D space delimited by the sample dimensions L_X, L_Y and L_Z. In both regular and random capillary network cases, the connections between nodes, when present, become capillaries.

Our simplified capillary networks are built by iterating over the following steps until the porosity of the synthesized network is within a predefined margin from that of the original. In a first step, the coordination number of each node is assigned by choosing from the probability distribution of the original capillary network (see Fig. S6a and Fig. S7a for examples of coordination number probability distribution in a regular and a random capillary network, respectively, overlaid with the distribution of the original sample). Then, capillaries connected to a node are deleted if needed to match the assigned coordination number. In a third step, capillary diameters are assigned by randomly choosing from the diameter probability distribution of the original capillary network (see Fig. S6b and Fig. S7b for examples of capillary diameter probability distribution in a regular and a random capillary network, respectively, overlaid with the distribution of the original sample). The final step in each iteration involves calculating the porosity of the simplified network by dividing the capillary volume by the sample volume and comparing it to the porosity of the original rock sample. This algorithm guarantees that the simplified network preserves the following properties: (i) porosity; (ii) capillary diameter distribution; and (iii) node coordination number distribution. Examples of 2D simplified capillary networks in regular and random configurations can be seen in Fig. S6c and Fig. S7c, respectively. Examples of 3D simplified capillary networks in regular and random configurations can be seen in Fig. S6d and Fig. S7d, respectively.

Several methodologies have been proposed to generate stochastic pore network models[38–40]. Although some approaches considered relevant geometrical and topological properties of pore space, such as pore-size, throat-size and coordination number distributions, two-phase flow simulation results on the networks generated have shown poor accuracy as compared to those of the original sample[38]. The Pore Network Model separates the pore space into spherical pores and cylindrical throats, which places limitations to accurately approximate the intricate pore geometry. Our statistically sampled capillary networks (ssCN), on the other hand, are built from the more accurate capillary network representation of the pore space[24] (see section S2), achieved through a sequence of short cylinders with gradually changing radii matching the local pore geometry extracted from the microtomography. As the ssCN built from this CNM preserves geometrical properties of the rock morphology relevant to fluid flow, namely porosity, capillary diameter distribution, and node coordination number distribution, it can more accurately approximate the original. Below, we assess the fidelity of the approximation against permeability measurements. The impact of these improvements on the accuracy of CO_2 drainage saturation, however, is limited due to the difficulty to compute two-phase flow in the original rock CNM.

Single-phase permeability calculations on ~50 different network configurations optimized

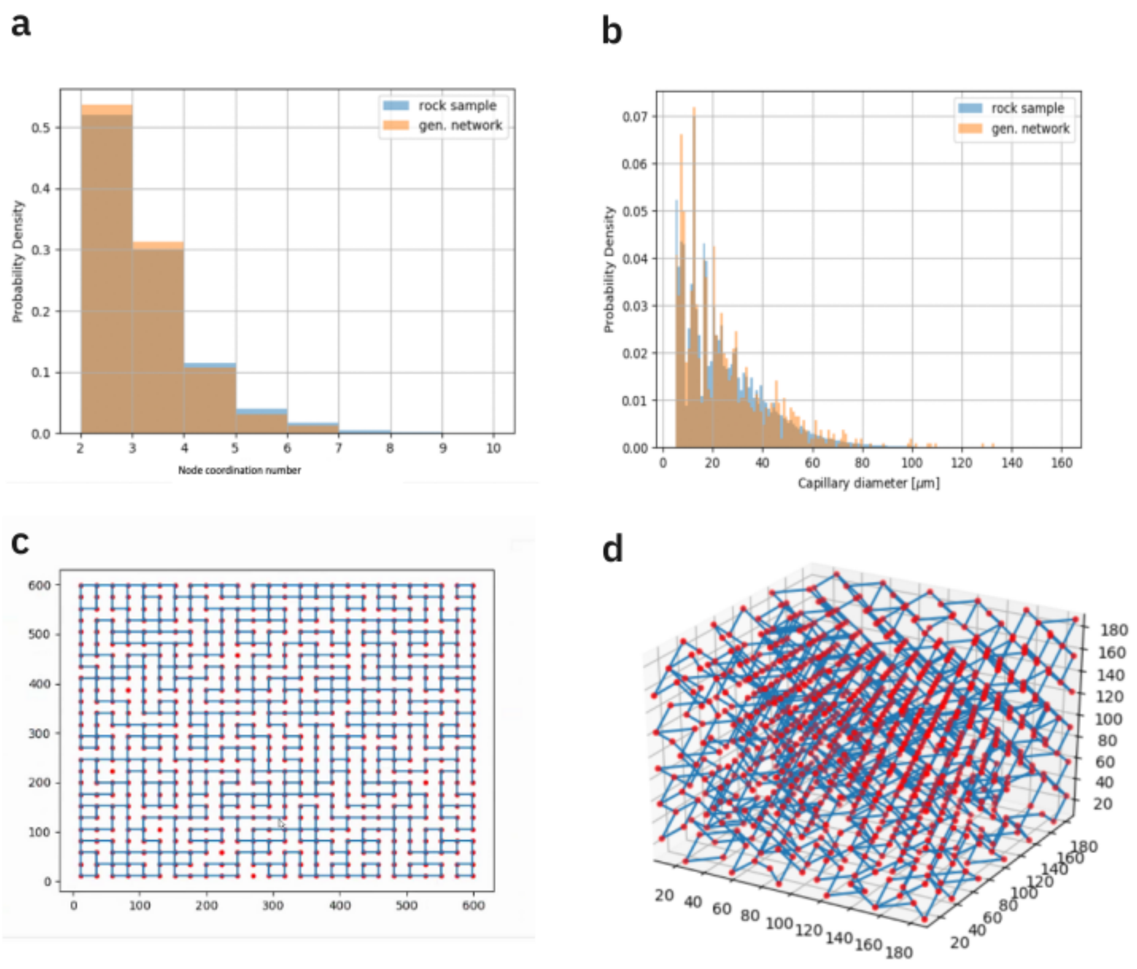

Supplementary Figure S6: **Regular simplified capillary network.** a) Node coordination number probability distributions and b) capillary diameter probability distribution of the synthesized network and the network of the original rock sample. c) 2D regular simplified capillary network with the capillaries represented in blue lines and the nodes in red dots. d) 3D regular simplified capillary network.

to represent the original Berea sandstone sample are shown in Fig. S8. The flow was imposed by applying an external 10 kPa/m pressure gradient along each axis. The simplified network optimization routine does not take into account the value of the permeability in the feedback loop. Potentially higher fidelity in the permeability value could be obtained by optimizing against this variable, however it requires running single phase simulations after each iteration, thus increasing the computational needs. The enhancements would also require an understanding of the influence of the pore geometry on the rock permeability in all X,Y,Z directions, as permeability of the

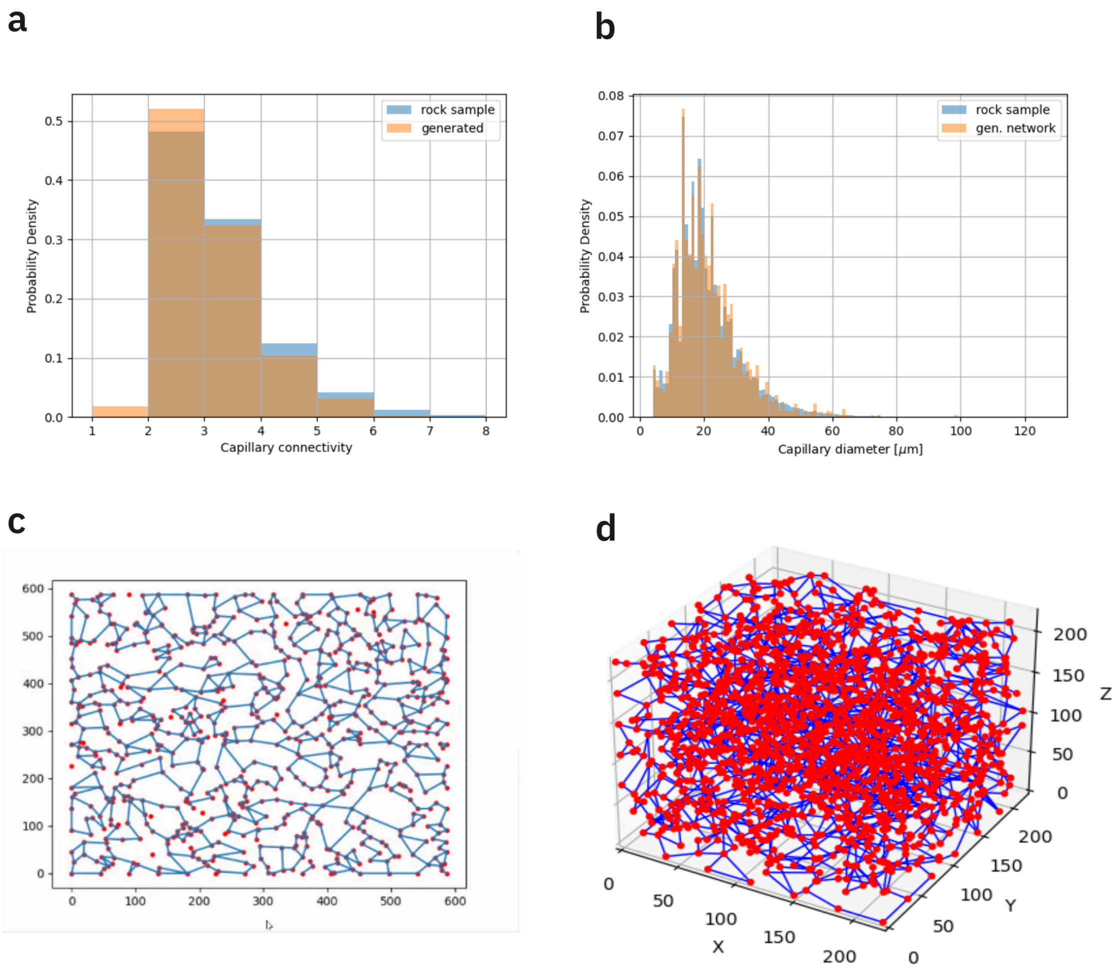

Supplementary Figure S7: **Random simplified capillary network.** a) Node coordination number probability distributions and b) capillary diameter probability distribution of the synthesized network and the network of the original rock sample. c) 2D random simplified capillary network with the capillaries represented in blue lines and the nodes in red dots. d) 3D random simplified capillary network.

original sample may not by isotropic. These additions to the optimization routine are certainly important enhancements to be included in future extensions to the code. Nevertheless, agreement within 2X of the permeability value ofthe original rock sample can be considered as good agreement.[54] From these results, we conclude that a network size of 1500 capillaries, about 0.5% of the total number of capillaries in the original sample, represents a good trade-off between accuracy and computational cost, with an average permeability within $\pm 3\sigma$ of the original. We observe that larger representations with more capillaries do improve precision but not the accuracy of the

average estimate.

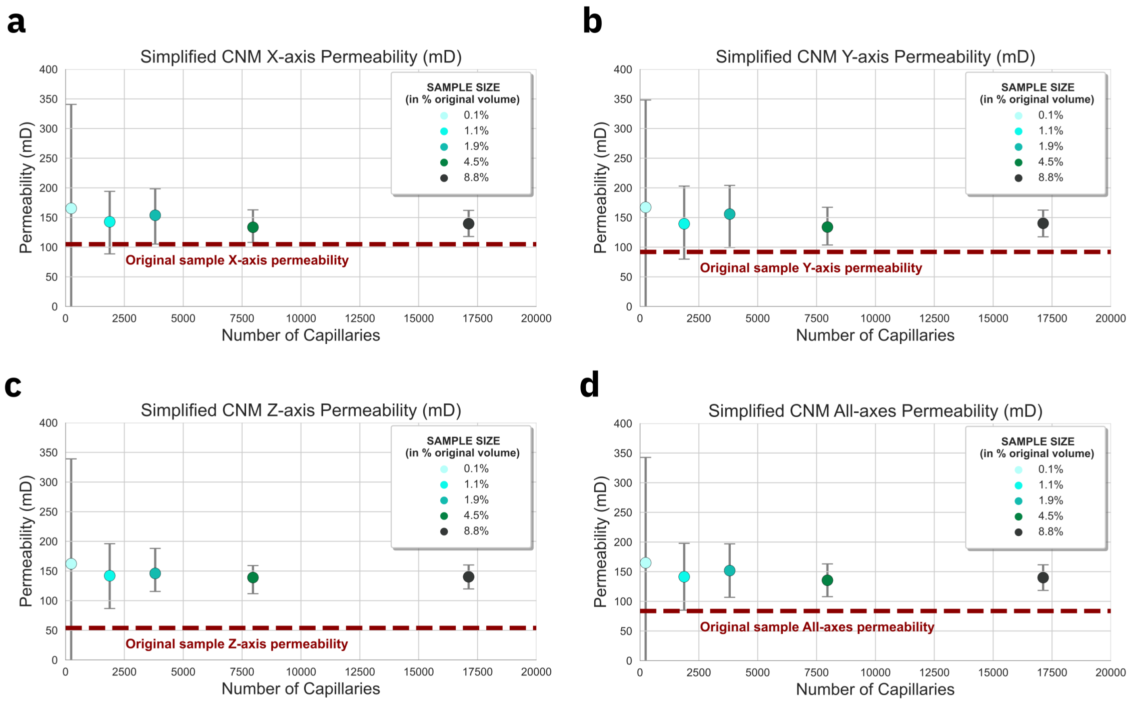

Supplementary Figure S8: **Permeability vs ssCN Size.** Mean permeability over the ensemble of ~ 50 simplified random network configurations along the (a) X-axis, (b) Y-axis, (c) Z-axis and (d) the mean across all three axes, as a function of the number of capillaries per network.

S6 Pressure Distribution Within Capillary Network

The resulting distribution of pressures after simulating the injection of $scCO_2$ into one of the simplified capillary networks is plotted in Fig. S9 under varying conditions of externally applied pressure gradient and fluid interface contact angle. This pressure distribution is the result of the interplay between the driving pressure gradient and the internal viscous and capillary forces in each capillary as described in section S4. For the range of capillary diameters shown in Fig 1b, capillary pressures within the network are estimated to be around 4 kPa for contact angles between 20° and 80°, as per the second term of right hand side of Eq. 3. We observe in Fig. S9a that a driving pressure gradient around 1×10^4 Pa/m is not sufficient to overcome the capillary pressures within the network for a contact angle of 20°. As a consequence, the fluid flow in this low pressure gradient regime is mostly driven by capillary pressure which tends to produce low CO_2 saturation.

For stronger externally applied pressure gradients, we observe a significant change in the pressure distribution of Fig. S9a and S9b towards higher pressures for both 20° and 90° contact angles. As the pressures induced by the external driving force are now sufficient to overcome the

opposing viscous and capillary forces, even under conditions of low contact angles, it is thus able to unplug many capillaries and reach higher saturation values. For the flow condition depicted in Fig. S9a, namely, 1×10^7 Pa/m pressure gradient and $20°$ contact angle, we obtained saturation values of 20% to 40%. But, more strikingly, for the condition represented in Fig. S9b, *i.e.*, 1×10^7 Pa/m pressure gradient and $90°$ contact angle, we obtained saturation values reaching 80%.

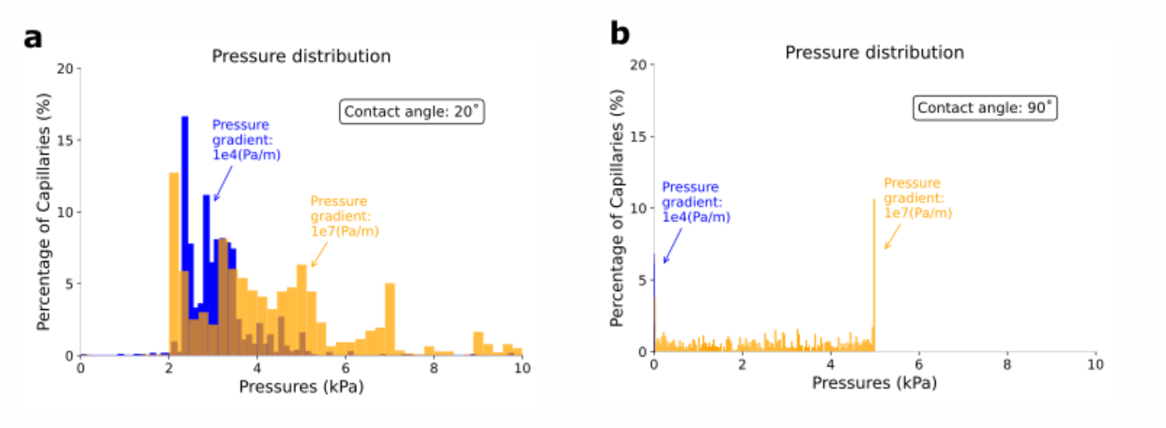

Supplementary Figure S9: **Pressure distributions within the CNM** Distribution of resulting pressures at the ends of capillaries for (a) contact angle $20°$, and pressure gradients 1×10^4 Pa/m and 1×10^7 Pa/m; (b) contact angle $90°$ and pressure gradients 1×10^4 Pa/m and 1×10^7 Pa/m.

S7 Saturation vs Injected Volume

Fig. S10a shows an example of supercritical CO_2 saturation as a function of time in the sandstone rock sample under study at a temperature of 473 K and an external pressure gradient of 5×10^6 Pa/m for a range of contact angles. The curves and shaded areas in the plot represent the mean and standard deviation of the simulations performed in an ensemble of ~ 50 ssCN, along the X, Y and Z axis. Alternatively we can display the saturation as a function of the $scCO_2$ injected volume (in units of "pore volume"), as shown in Fig. S10b for the same simulation results. To reduce the influence of backward flow, the injected volume in this context is computed from the sum of the $scCO_2$ saturation at each time step plus the volume of $scCO_2$ ejected from the outlet capillaries, calculated by integrating over time the product of the outlet capillaries cross sectional area and the local flow speeds, normalized by the total pore volume occupied by all capillaries in the network.

Fig. S10b shows that for most contact angles, the saturation of $scCO_2$ reaches a plateau, indicating that fluid is not being retained within the pore space. As a measure of the injection efficiency, we define the variable *Weighted Saturation* (wS) as the measured saturation (S) scaled by the ratio of saturation to injected volume (IV), that is, $wS = S\frac{S}{IV}$, with units of pore volume of injected CO_2. As the saturation plateaus, the value of the wS peaks and any additional injection does not result in further permeation of the pore space with potential for capillary trapping. This variable thus emphasizes the effect that the CO_2 injected volume has in the maximum achievable

saturation, as observed in Fig. S10c (for a range of pressure gradients, with fixed 85° contact angle) and Fig. S10d (for a range of contact angles, with fixed 5×10^6 Pa/m pressure gradient).

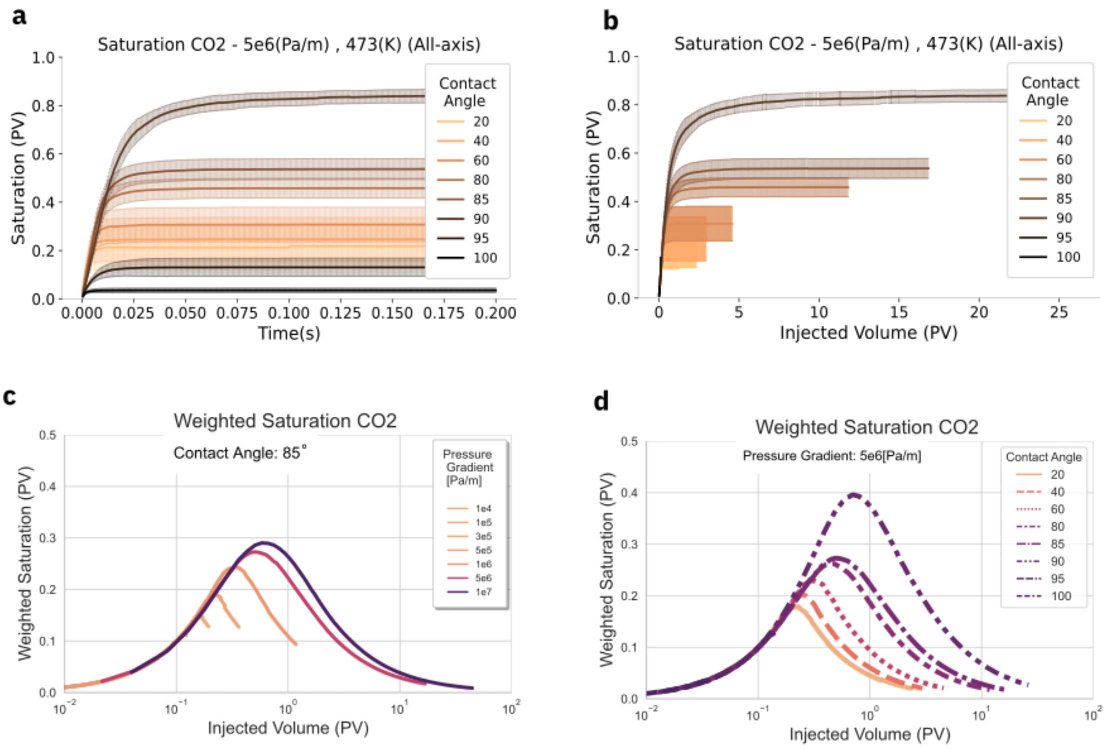

Supplementary Figure S10: **Weighted Saturation *vs.* Injected Volume**. Outcome of two-phase simulations measured as the mean and standard deviation of the results from an ensemble of 50 ssCN, assuming supercritical CO_2 as injected fluid, water as resident fluid, a temperature of 473 K and an applied pressure of 1×10^6 Pa/m. (a) Saturation of supercritical CO_2 as a function of time for various values of contact angle. (b) Saturation as a function of injected volume across various values of contact angle. (c) Weighted saturation as a function of injected volume for a fixed contact angle of 85° and a range of applied pressure gradients, and (d) weighted saturation as a function of injected volume for a fixed applied pressure of 5×10^6 Pa/m and the range of contact angles.

S8 Simulation Toolkit for Scientific Discovery (ST4SD)

In our study we scanned through 4 temperature scenarios, studied 8 fluid-interface contact angles per scenario and no less than 8 different driving pressure gradient cases per angle, totaling 256 different injection conditions to be simulated. Per injection condition, 150 flow simulations were executed applying driving pressure along all three axes on each of the 50 simplified capillary networks in the ensemble, requiring proper parsing and aggregation of nearly 40,000 simulations. To manage the large parameter space, we leveraged high-throughput automated simulation workflows. In particular, we employed the Simulation Toolkit for Scientific Discovery (ST4SD)[41] to automate

the execution of long simulation campaigns with several chained steps. The use of such workflow scheduler ensures the reproducibility of our results and enable efficiency gains by optimising the use of computing resources.

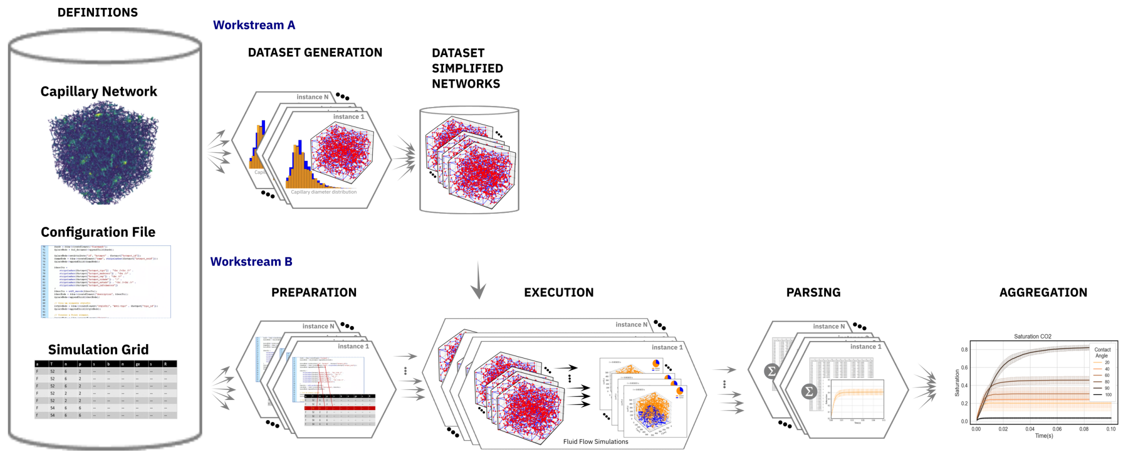

Supplementary Figure S11: **Schematic ST4SD workflow.** Automated computational methodology showing, from left to right, experiment definition, preparation of inputs, simulation execution, output parsing, and aggregation of results

Fig. 1 illustrates the conceptual workflow and Fig. S11 shows the sequence of steps executed in an ST4SD experiment. A CNM representation of a rock sample is used as input to the ST4SD routine. This capillary network model is, however, too detailed to solve numerically in a two-phase flow scenario, so we generate tens of simplified capillary networks that meaningfully represent the properties of the original network (see Supplementary Section S5). Each simplified capillary network is then used as input to independent flow simulations that will estimate relevant physical properties in each representative network. Finally, the individual results from each simplified network are aggregated and combined into a single estimate that applies to the reference network.

Fig. S11 shows two connected workstreams. Workstream *A* refers to the process of generating a large ensemble of simplified capillary networks from that of a high-resolution digital rock sample. The Dataset Generation step follows the methodology described in Supplementary Section S5. Taking the original rock sample CNM as input, the workflow launches in parallel many processes to generate simplified capillary networks. In each parallel process, the algorithm alternates between molding a (initially random) set of connected capillaries into matching the morphological properties of the reference rock, and running single-phase flow simulations to assess the permeability until a convergence criterion is reached. The outcome of this workstream is an ensemble of simplified capillary networks whose morphological properties mimic those of the original network.

Workstream *B* refers to the simulated injection of $scCO_2$ on an ensemble of simplified capillary networks and extracting relevant properties from the aggregate of the results. A simulation

parameter grid containing all the values to be executed is used as input to the *preparation* step of workstream *B*. Per instance of this simulation grid, the values of parameters such as applied pressure, temperature or contact angle are inserted into the configuration files of the simplified capillary networks produced in workstream *A*. In the *execution* step, two-phase fluid flow simulations are executed for the ensemble of networks and the interfaces are tracked within the capillaries to extract saturation values as a function of time. During *parsing* the saturation of all networks are averaged as a function of time and injected volume, and passed to the *aggregation* step where they are saved together with the results from other instances.

S9 Injection Safety and Efficiency

Fig. 3 of the main manuscript explores the efficiency and security of the $scCO_2$ drainage process in deep reservoirs at a fixed 473 K temperature. Fig. S12 explores the safety of the process by plotting the value of saturation at 90% of the maximum *vs.* the injected volume required to reach that point. Injected volumes beyond 1 PV indicate that some of the $scCO_2$ was not retained within the sample, representing a leakage concern. We observe larger values of injected volume required to achieve similar levels of saturation with lower temperatures. Fig. S13 explores the efficiency of the injection process by plotting the maximum weighted saturation of $scCO_2$ as a function of injected volume, representing the largest achievable saturation volume without $scCO_2$ breakthrough. Data points closer to the diagonal represent maximum injection efficiency, and this trend improves with higher temperatures.

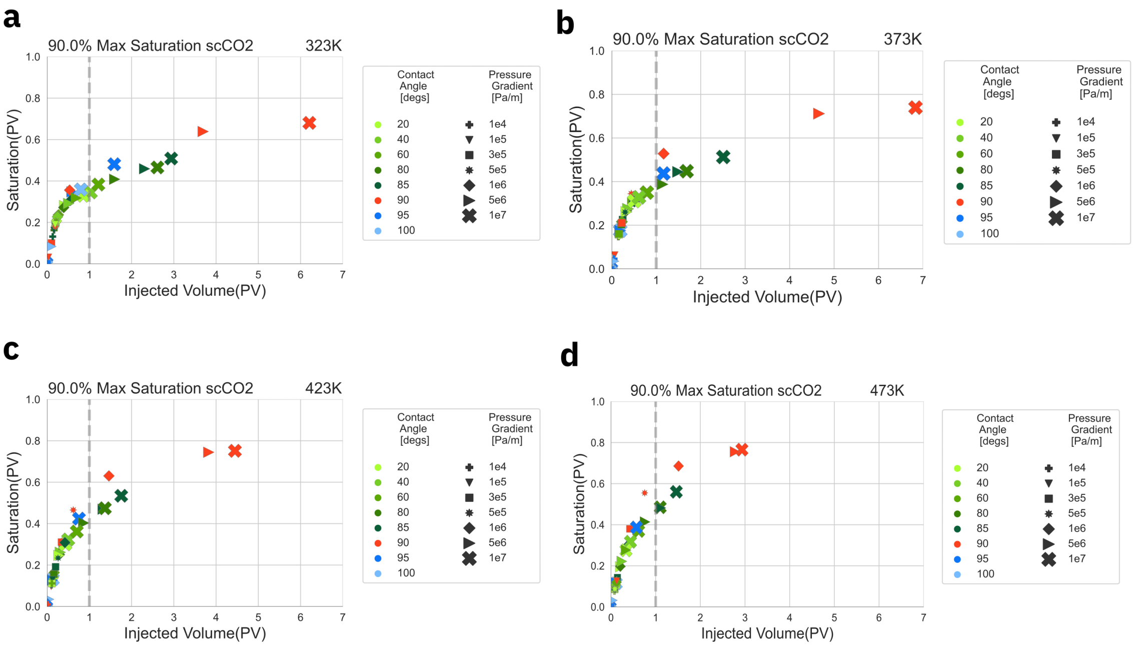

Supplementary Figure S12: **Value at 90% maximum saturation *vs.* injected volume.** Green colors represents the CO_2-wet regime, red shows the zero capillary pressure regime and blue represents the intermediate-wet regime. Larger marker sizes represent higher pressure gradients at temperatures a) 323 K, b) 373 K c) 423 K and d) 473 K.

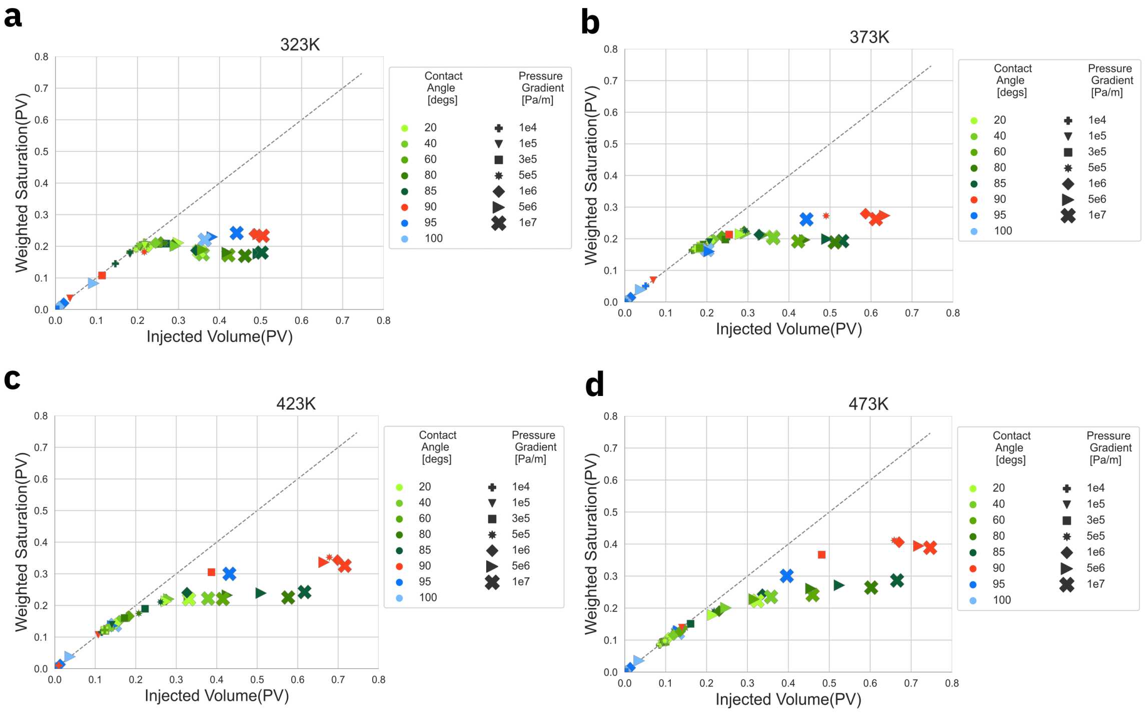

Supplementary Figure S13: **Maximum weighted saturation vs injected volume.** Green colors represents a CO_2-wet regime, red shows the case of zero capillary pressure and, in blue, the intermediate-wet regime. Larger markers represent higher pressure gradients at temperatures a) 323 K, b) 373 K c) 423 K and d) 473 K.
